# Barriers and facilitators to HIV prevention interventions for reducing risky sexual behavior among youth worldwide: a systematic review

**DOI:** 10.1186/s12879-022-07649-z

**Published:** 2022-08-08

**Authors:** Fungai Mbengo, Esther Adama, Amanda Towell-Barnard, Arvin Bhana, Maggie Zgambo

**Affiliations:** 1 School of Nursing and Midwifery, Edith Cowan University, Joondalup, WA 6027 Australia; 2 Health Systems Research Unit, South African Medical Research Council, Tygerberg, 7505 South Africa; 3 Centre for Rural Health, School of Nursing and Public Health, University of KwaZulu-Natal, Durban, 4041 South Africa

**Keywords:** Barriers, Facilitators, HIV prevention intervention, Risky sexual behavior, Youth, Systematic review

## Abstract

**Background:**

Interventions aimed at reducing risky sexual behavior are considered an important strategy for averting Human Immunodeficiency Virus (HIV) infection among youth (15–24 years) who continue to be at risk of the disease. Enhancing intervention success requires a comprehensive understanding of the barriers and facilitators to interventions targeting youth. However, there is lack of a systematic review of both quantitative and qualitative studies to comprehensively identify and synthesize barriers and facilitators to HIV prevention interventions for reducing risky sexual behavior among youth worldwide. This review aimed to identify and synthesize barriers and facilitators to HIV prevention interventions for reducing risky sexual behavior among youth globally based on original peer-reviewed studies published in the last decade.

**Methods:**

The Joanna Briggs Institute approach for mixed methods systematic reviews and Preferred Reporting Items for Systematic Reviews and Meta-Analysis guidelines were used to guide this review. Nine electronic databases, Joint United Nations Programme on HIV/AIDS and World Health Organization websites, and reference lists of included studies and systematic reviews on barriers and facilitators to HIV prevention interventions for reducing risky sexual behavior among youth were searched for eligible articles. Studies that met the inclusion criteria underwent quality appraisal and data extraction. Findings were analyzed using thematic synthesis and underpinned by Nilsen, 2015’s Determinant Framework.

**Results:**

Overall 13 studies comprising of eight qualitative studies, four quantitative studies and one mixed methods study were included in the review. Several barriers and facilitators across the five Determinant Framework domains were identified. Most of the barriers fell under the characteristics of the context domain (e.g., gender-biased norms). The next important group of barriers emerged within the characteristics of the end users domain (e.g., fear of relationship breakdown). In terms of facilitators, the majority fell under the characteristics of the strategy of facilitating implementation domain (e.g., implementation of intervention with fidelity) and characteristics of the end users domain (e.g., fear of pregnancy or sexually transmitted infections). The next common set of facilitators appeared within the characteristics of the context domain (e.g., family support).

**Conclusion:**

This review identified several multi-level barriers and facilitators to HIV prevention interventions for reducing risky sexual behavior among youth. Multi-level and combination approaches are needed to address these factors and enhance intervention success.

**Supplementary Information:**

The online version contains supplementary material available at 10.1186/s12879-022-07649-z.

## Background

Young people continue to be at considerable risk for Human Immunodeficiency Virus (HIV) despite intervention efforts to control the disease [1]. Specifically, youth aged between 15 and 24 years account for nearly 30% of all new HIV infections and 9% of all people living with HIV worldwide [2]. Moreover, there are indications that HIV infection rates among youth will increase annually by 13% leading to approximately 3.5 million new infections by 2030 [2, 3]. The socio-ecological model [4, 5] suggests that young people are vulnerable to HIV due to numerous factors at different socio-ecological levels that affect risky sexual behaviors including low self-esteem, lack of parent–child communication, peer pressure, poverty [6, 7], alcohol or drug abuse [8], limited HIV-related knowledge, gender-based violence [9], gender disparities and cultural factors [10, 11].

To date HIV has no cure, therefore HIV prevention programs aimed at reducing risky sexual behavior are regarded as an important strategy of controlling the disease. As a result, various HIV prevention interventions for reducing risky sexual behavior among youth have been developed [12, 13]. Reviews that have evaluated the efficacy of these interventions suggest that such strategies are more effective at changing non-behavioral outcomes (e.g., attitudes, beliefs, intentions and HIV-related knowledge), and less effective at changing behavioral outcomes (e.g., condom use) [14–16].

Developing effective strategies to enhance intervention efficacy requires a comprehensive understanding of the barriers and facilitators to HIV prevention interventions targeting youth. Theoretical framework is valuable for identifying barriers and facilitators to intervention and for developing policies and strategies to promote intervention success [17, 18]. One such theoretical framework is the Determinant Framework proposed by Nilsen, 2015 [17]. This framework is composed of five major domains or levels, which can capture a myriad of factors that affect the success of an intervention. These domains include: (i) characteristics of the implementation object, which involves features of an intervention that might influence intervention success (e.g., duration of the intervention); (ii) characteristics of the users or adopters, which includes features of implementers of an intervention that might influence intervention success (e.g., training, skills and experience of the implementers of an intervention); (iii) characteristics of the end users, which includes attributes of clients or recipients of an intervention that might affect intervention success (e.g., age, knowledge and self-efficacy of the recipients of an intervention); (iv) characteristics of the context, which involves attributes of conditions or surroundings of an intervention that might affect intervention success (e.g., availability of funding or resources within the organization implementing the intervention); and (v) characteristics of the strategy of facilitating implementation, which includes tactics of delivering a program that might influence intervention success (e.g., implementation of intervention with fidelity or according to plan) [17].

In the last decade, a number of systematic reviews have been conducted on barriers and facilitators to HIV prevention interventions for reducing risky sexual behavior in youth [19–25]. However, these reviews have synthesized research findings from studies that were conducted using quantitative approaches [19–25]. Furthemore, these reviews have focused on studies conducted in a specific geographical region; for instance, Sub-Saharan Africa [22–24], developing countries [20], Europe [21], and South Africa [19, 25]. Other reviews have focused on barriers [22] or facilitators [20] only. Moreover, in the last decade, no systematic review has included both quantitative and qualitative studies to identity and synthesize barriers and facilitators to HIV prevention interventions for reducing risky sexual behavior among youth worldwide. Therefore, the purpose of this review was to synthesize current global evidence on barriers and facilitators to HIV prevention interventions for reducing risky sexual behavior among youth based on peer-reviewed quantitative, qualitative or mixed methods studies published in the last decade. Conducting this review was essential to provide holistic evidence to support youth-focused HIV prevention programs across the globe. Consistent with other reviews, barriers were defined as factors that impede intervention success (e.g., lack of funding) [23]. Facilitators were defined as factors that promote intervention success (e.g., availability of funding) [23].

## Methods

### Study design

This systematic review was conducted using the Joanna Briggs Institute (JBI) approach for mixed methods systematic reviews [26] and Preferred Reporting Items for Systematic Reviews and Meta-Analysis (PRISMA) guidelines [27]. The mixed methods approach was employed to obtain a comprehensive synthesis of evidence than that provided by a single method approach [28]. The protocol for this review was registered in PROSPERO (registration number CRD42020187272) [29].

### Inclusion criteria

Studies were considered in this review if they:

- were original peer-reviewed studies written in English language;
- included youth (15–24 years) as intervention recipients;
- presented barriers and/or facilitators to HIV prevention intervention for reducing risky sexual behavior;
- were conducted in any geographical location;
- were conducted using quantitative, qualitative or mixed methods study designs;
- were published between January 2010 and April 2022. This period was chosen to obtain current evidence.

### Search strategy

The Cambridge Core, CINAHL, Cochrane Library, Google Scholar, MEDLINE, ProQuest Central, PsycINFO, Oxford Journals and Web of Science datababes, Joint United Nations Programme on HIV/AIDS (UNAIDS) and World Health Organization (WHO) websites were searched for eligible studies using relevant keywords. Moreover, reference lists of included studies and systematic reviews on barriers and facilitators to HIV prevention interventions for reducing risky sexual behavior among youth were screened to identify additional eligible studies. Keywords used to search included “Youth”, “Barriers”, “Facilitators”, “HIV Prevention Intervention”, “Risky Sexual Behavior”, “Efficacy” and “Implementation” (Additional file 1: Table S1). Booleans (e.g., “OR” and “AND”) were used to combine similar and different search terms, respectively. For example, (youth OR young people OR teen OR young adults OR students OR adolescents) AND (barriers OR challenges OR constrains OR difficulties OR obstacles OR issues OR problems). A detailed search strategy is shown in Additional file 1: Table S1 using MEDLINE as a example. The search strategy was adapted to other databases/websites.

### Study selection

Following the search, all identified references were collated and uploaded into EndNote X9 Reference Management System and duplicates removed. FM, EA and ATB assessed the title, abstract and full-text articles against the inclusion criteria. Any disagreements were resolved through consensus.

### Assessment of methodological quality

The methodological quality of the studies were assessed independently by FM, EA and ATB, and differences were resolved through discussion. Authors of papers were contacted to request missing information. Quantitative studies were assessed using the JBI Critical Appraisal Checklist for Randomized Controlled Trials (13 items) [26] and JBI Critical Appraisal Checklist for Quasi-Experimental Studies (nine items) [26]. The JBI Critical Appraisal Checklist for Qualitative Research (10 items) [26] was used to assess qualitative studies. Mixed methods studies were appraised using both methods. A study was considered ‘good’ if it scored more than 70%, ‘fair’ if it scored between 50 and 70% and ‘poor’ (excluded) if it scored less than 50% on the quality appraisal tool.

### Data extraction

FM extracted data from the included studies using a standardized JBI data extraction form [26]. Data extraction was discussed with EA, MTB and AB, and any discrepancies were resolved though consensus. Details extracted include: author, year of publication, title of the study, purpose or objective of the study, study location, setting, study design, population and sample size, methods, data analysis and findings (barriers and facilitators). Findings regarding barriers and facilitators were identified through repeated reading of the studies. Additional file 2: Table S2–S14 presents extracted findings relating to barriers and facilitators with each finding supported by an illustration from the same text that informed the finding. As recommended by JBI, quantitative data were converted into ‘qualitized data’ (textual descriptions or narrative interpretation of the quantitative findings) to enhance integration with qualitative data.

### Data synthesis

Synthesis involved two phases. In the first phase, a thematic synthesis approach was used [30, 31]. This involved reading of the findings several times for familiarization, systematic coding of data and identification of prominent themes. In the second phase, barriers and facilitators obtained from thematic synthesis were mapped to the Determinant Framework suggested by Nilsen, 2015 [17] to highlight the key barriers and facilitators, and strategies needed within the different Determinant Framework levels to improve intervention success [17, 18]. Synthesis was undertaken by FM, and discussed with EA, MTB and AB. Any disagreements were resolved through consensus.

## Results

### Search outcome

**Fig. 1:**
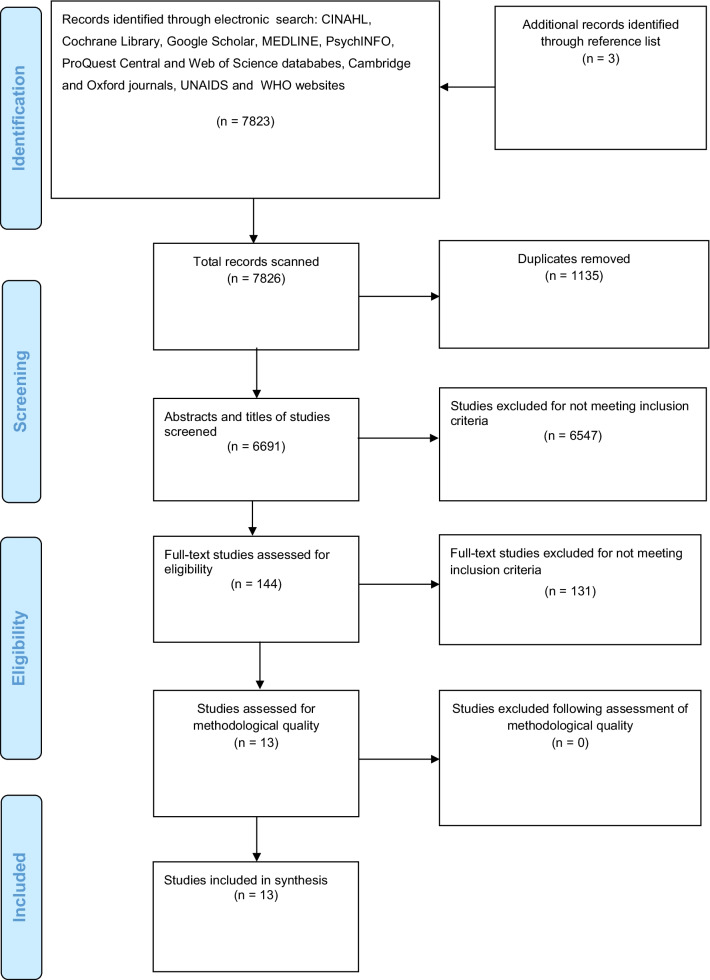
PRISMA flow diagram illustrating selection of studies

Of the 7826 records identified, 6691 titles and abstracts were screened. Of these, 144 full-texts were further screened and 13 studies met the inclusion criteria. No studies were excluded following assessment of methodological quality (Fig. 1).

### Characteristics of the included studies

The characteristics of the included studies are summarized in Table 1. Studies included in the review consisted of eight qualititative studies [32, 36–39, 42–44], four quantitative studies [34, 35, 40, 41] and one mixed methods study [33]. Of the quantitative studies, two studies were quasi-experimental studies without a comparison group [34, 35], one study was a quasi-experimental study with a comparison group [40], and one study was a randomised controlled trial [41].

**Table 1:** Characteristics of included studies

Author, year & title	Study purpose/objective	Study location	Setting	Study design	Population/sample size	Methods	Data analysis
Al-iryani et al. 2011 [32]	To highlight the factors that facilitated or inhibited community based peer education	Yemen	Community setting (4 poor and vulnerable areas)	Qualitative study design	16 community focal points, 21 peer educators, 10 targeted young people in communities, 3 young female sex workers, 2 local council members	Focus group discussions and in-depth interviews	Thematic analysis
Aung et al. 2017 [33]	To evaluate the effectiveness and acceptability of integrated, community-based, and clinic-based intervention	Myanmar	Community and healthcare setting (3 townships, clinics and drop-in centers)	Mixed methods study design; qualitative component	54 young men who have sex with men aged 15–24 years and 18 peer educators	Focus group discussions	Thematic analysis
Garofalo et al. 2012 [34]	To assess the efficacy, feasibility and acceptability of Life Skills intervention	United States of America	Community setting (1 urban geographical area, night clubs, pageants, local parks, youth centre, community organization)	Quasi-experimental one group, before-after design	51 Transgender women aged 16–24 years	Audio-computer-assisted self-interviewing technology	Descriptive analysis and statistical analysis
Greene et al. 2016 [35]	To assess the effectiveness and acceptability of online HIV prevention intervention	United States of America	Community setting (local non-profit community-based organization)	Quasi-experimental one group, before-after design	343 ethnically and racially diverse young men who have sex with men aged 18–24 years	Online evaluation surveys	Descriptive analysis and statistical analysis
Jewkes et al. 2010 [36]	To explore how participants made meaning from the Stepping Stones intervention and how it influenced their lives	South Africa	Community setting (poor, rural formerly subsistence farming area)	Qualitative study design	11 women and 10 men majority aged 17–21 years, and 4 focus groups	In-depth interviews and focus group discussions	Content analysis and analytic induction
Morrison-Beedy et al. 2013 [37]	To describe the experiences of adolescent girls who participated in a sexual risk‐reduction intervention	United States of America	Community setting (poor urban area, community-based organization)	Qualitative descriptive study design	26 African American urban, low income girls aged 15–19 years	Semi-structured interviews	Thematic analysis
Musiimenta., 2012 [38]	To identify contextual mediators that influence the youth’s decision to adopt and maintain the HIV/AIDS preventive behavior advocated by a computer-assisted intervention	Uganda	Educational setting (secondary schools)	Qualitative study design	20 youth	Individual telephone interviews	Grounded theory’s three-stage coding process analysis
Ridgeway et al. 2020 [39]	To explore individual, interpersonal- and household-level factors influencing HIV-related sexual risk behavior among adolescent girls who participated in the Women First intervention	Mozambique	Community setting (poor rural area)	Qualitative study design	28 adolescent girls aged 13–19 years; mean age 16 years, 30 household heads and 53 influential men	In-depth interviews	Primary analysis, inductive approach and within-case and cross-case comparative analysis
Rohrbach et al. 2019 [40]	To assess effectiveness and implementation of the HIV/STI/Pregnancy prevention program	United States of America	Educational setting (24 urban public middle schools)	Quasi-experimental non-equivalent two-group, before-after study design	44 teachers and trained program staff	Implementation logs and observations	Analysis not reported
Sales et al. 2012 [41]	To examine factors associated with adolescents’ failure to improve their condom use behaviors after participating in an STI/HIV prevention intervention	United States of America	Healthcare setting (3 downtown clinics)	Randomized controlled trial design	205 African-American adolescent females aged 15–21 years; mean age 17.9 years	Audio computer assisted self-interview and self-collected vaginal swab	Descriptive analysis, statistical analysis and logistic regression
Sales et al. 2012a [42]	To identify factors associated with young African American females’ lack of increase in condom use post-participation in an HIV prevention intervention	United States of America	Healthcare setting (2 downtown sexual health clinics)	Qualitative grounded theory study design	50 young African American women aged 18–23 years; mean age 20.5 years	Semi-structured interviews	Grounded theory’s three-stage coding process analysis
Wamoyi et al. 2012 [43]	To explore young people’s memories and views of the relevance of the sexual and reproductive health intervention and their ability to apply what they had learned 7–9 years after exposure to the intervention	Tanzania	Community setting (rural area)	Qualitative study design	23 rural Tanzanian young people males aged 24–29 years and females aged 24–30 years	In-depth interviews	Preliminary analysis and grounded theory’s three-stage coding process analysis
Wight et al. 2012 [44]	To explain the *MEMA kwaVijana* trial outcomes	Tanzania	Community, educational and healthcare setting (rural area, communities, primary schools, health facilities)	Qualitative ethnography study design	92 trial participants, 6 single sex groups of young villagers and 9 villages	Participant observation, in-depth interviews, focus group discussions, stimulated patient visits, internal monitoring and evaluation, and annual surveys of implementers	Thematic analysis

Of the included studies, six were published in 2012 [34, 38, 41–44]. Seven studies were published each in 2010 [36], 2011 [32], 2013 [37], 2016 [35], 2017 [33], 2019 [40] and 2020 [39].

Seven countries were represented across the included studies including the United States of America [34, 35, 37, 40–42], Tanzania [43, 44], Yemen [32], Myanmar [33], South Africa [36], Uganda [38] and Mozambique [39]. As per the World Bank definition [45], majority of these countries (n = 6) were low/middle-income countries, except the United States of America (a high-income country). Four of the low/middle-income countries were from Sub-Saharan Africa, a region with the highest number of people living with HIV than any other region in the world [2].

In terms of setting, seven studies were conducted in a community setting [32, 34–37, 39, 43], two studies were undertaken in an educational setting [38, 40], two studies were conducted in a healthcare setting [41, 42], one study was undertaken in community and healthcare setting [33], and one study was conducted in the community, educational and healthcare setting [44].

Participants were youth or intervention recipients in eight studies [34–38, 41–43]. One study included youth and implementers [33], one study involved youth, implementers and community members [32], one study included youth, family members and community members [39], one study incorporated youth and community members [44]. One study involved implementers but was included because the intervention recipients were youth [40]. The number of participants within the included studies ranged from 20 to 343. Rorhbach, 2019 [40] did not report the number of implementers (trained program staff). The age of youth participants ranged from 15–24 years in eight studies [33–37, 39, 41, 42]. Three studies did not mention the age of youth participants [32, 38, 44]. One study reported the age of youth participants as ranging from 24 to 29 years for males and 24–30 years for females but was included because participants were aged 15–24 years when the intervention was implemented [43].

Overall, the quality of the included studies was good with nine studies [32, 34, 35, 39–44] scoring more than 70% on the quality appraisal tool. Four studies [33, 36–38] were rated as fair (scored between 50 and 70% on the quality appraisal tool) [Additional file 1: Table S2–S4].

### Barriers and facilitators

Thematic synthesis [30, 31] and mapping of the study findings to the Determinant Framework [17] highlighted a wide range of barriers and facilitators to HIV prevention interventions for reducing risky sexual behavior among youth. A summary of the identified barriers and facilitators across the five Determinant Framework domains is presented in Table 2.

**Table 2:** Identified barriers and facilitators to interventions targeting youth across the five domains of Nilsen, 2015’s Determinant Framework [17]

Barriers	Facilitators
1. Characteristics of the implementation object	1. Characteristics of the implementation object
1.1 Barriers to intervention acceptability among youth -Incompatibility of intervention content with the needs of youth [33, 43] -Long duration of the intervention [33, 35] -Complexity of the intervention [35]	1.1 Facilitators to intervention acceptability among youth -Compatibility of intervention content with the needs of youth [35–37]
1.2 Barriers to intervention acceptability among community members -Incompatibility of intervention content with the needs of community members [44]	1.2 Facilitators to intervention acceptability among implementers -Relative advantage of the intervention [32] Total facilitators: 2
1.3 Barriers to youth’s participation in the intervention -Age requirements that excluded other youth [33] -Restricted days and times of the intervention [34]	
1.4 Barriers to risky sexual behavior reduction among youth -Limited intervention content (e.g., intervention content addresses individual factors such as knowledge without addressing structural factors such as poverty, unemployment) [32, 44] Total barriers: 7	
2. Characteristics of the users/adopters	2. Characteristics of the users/adopters
2.1 Barriers to intervention acceptability among youth -Adult/old implementers [37]	2.1 Facilitators to intervention acceptability among youth -Approachability/friendliness implementers [33, 37] -Experience of implementers [37]
2.2 Other barriers to intervention success -Implementers’ lack of knowledge related to intervention content [44] -Poor education or training of implementers [44] -Implementers’ lack of exemplary or positive behavior [44] Total barriers: 4	2.2 Other facilitators to intervention success -Training of implementers [32] -Implementers’ knowledge related to intervention content [44] Total facilitators: 4
3. Characteristics of the end users	3. Characteristics of the end users
3.1 Barriers to risky sexual behavior reduction among youth -Low perceptions of risk of sexually transmitted infections including HIV [33, 38, 42–44] -Fear of relationship breakdown [33, 38, 42, 43] -Desire for pregnancy/children [42, 43] -Being stubborn/hard hardheaded [42, 43] -Belief that one is incapable of change [42, 44] -Negative attitudes towards condom use [32, 44] -Poor decision-making skills [44] -Lack of self-confidence [42] -Concern for privacy [33] -Fear of side effects of contraceptives [44] -Having high sensation seeking [41] -Being under the influence of alcohol/drugs [42] -Being reliant on avoidance strategies [42] -Being unprepared [42] -Preferring not to adopt an HIV prevention method (e.g., condoms use) [43] -Limited sexual health knowledge [44] -Negative experiences associated with using contraceptives [43] -Desire to meet basic material needs [44]	3.1 Facilitators to risky sexual behavior reduction among youth -Fear of pregnancy/sexually transmitted infections including HIV [32, 37, 43] -Having strong ambitions/being future oriented [39, 44] -Being knowledgeable [42] -Having good problem-solving skills [42] -Having high self-confidence [42] -Intentions/readiness to change [36] -Negative experiences in a relationship [39] -Being self-reliant [39] -Having high self-motivation [39] -Having high self-respect [42] -Having high sense of responsibility [42] -Low socio-economic status (e.g., lack of money to pay for sex) [44]
3.2 Barriers to youth’s participation in the intervention -Concern for privacy [32, 44] -Fear of stigma [44]	3.2 Facilitators to youth’s participation in the intervention -Perceived benefits of the intervention [37] Total facilitators: 13
3.3 Other barriers to intervention success -Being stubborn/hardheaded/uncooperative [44] -Having limited knowledge (e.g., about the intervention [44] -Low literacy [44] Total barriers: 23	
4. Characteristics of the context	4. Characteristics of the context
4.1 Interpersonal	4.1 Interpersonal
4.1.1 Barriers to risky sexual behavior reduction among youth -Partner’s refusal to use an HIV prevention method (e.g., condom use, HIV testing [32, 33, 38, 39, 42] -Lack of financial support from the family [38, 39, 43] -Peer pressure [38, 43, 44] -Lack of child-parent communication on sexual issues [36, 38] -Controlling partner [42, 43] -Relationship issues (e.g., current boyfriend and unstable relationships) [41, 42] -Parent’s refusal of an HIV prevention method (e.g., HIV testing [32] -Violent partner [36, 41] -Partner’s negative attitudes towards condom use [38] -Poor role models [38] -Lack of restrictive parenting [38] -Partner suspect fidelity if the other partner request protected sex [42) -Partner’s desire for pregnancy [42] -Partner is under the influence of drugs [42] -Partner’s preferences not to adopt an HIV prevention method (e.g., condom use) [39]	4.1.1 Facilitators to risky sexual behavior reduction among youth -Partner’s consent to use an HIV prevention method (e.g., condom use [42, 43] -Family support [39, 44] -Restrictive parenting [38, 44] -Positive peer influence [38] -Teacher advice [38] -Parental advice [39] -Stable relationships [42] -Partner does not suspect fidelity if the other partner request protected sex [42] -Family/parental religious beliefs (e.g., raised in a family with religious beliefs against engaging in risky sexual behavior) [44]
4.1.2 Other barriers to intervention success -Lack of support for critical thinking among youth [44]	
4.2 Community	4.2 Community
4.2.1 Barriers to risky sexual behavior reduction among youth -Gender-biased norms [36, 38, 39, 43, 44] -Myths about contraceptives [32, 44] -Norms discouraging discussion of sexual issues between parents and children [32, 44] -Limited resources/services in the community (e.g. programs for youth, condoms) [32, 33] -Cultural beliefs [44]	4.2.1 Facilitators to risky sexual behavior reduction among youth -Norms encouraging healthy sexual behavior (e.g., abstinence and delaying of sexual debut) [44] -Religious beliefs discouraging risky sexual behavior [38]
4.2.2 Barriers to youth’s participation in the intervention -Violence in the community/neighborhood [34] -Incarceration [34]	
4.3 Organizational or institutional	4.3 Organizational or institutional
4.3.1 Barriers to risky sexual behavior reduction among youth -Limited resources (e.g., condoms, human resources) [44] -Inaccessibility of services (e.g., condoms, healthcare facilities) [44] -Poor quality of services (e.g., lack of confidentiality) [44]	4.3.1 Facilitators to intervention acceptability among youth -Accessibility and friendliness of the intervention venue [33] Total facilitators: 12
4.3.2 Barriers to youth’s participation in the intervention -Inaccessibility of intervention venue [34, 37]	
4.3.3 Other barriers to intervention success -Limited resources (e.g., financial and human resources) [44] -Restrictions on depicting of condoms in schools [44] -Poor quality of services (e.g. lack of confidentiality, inappropriate clinical advice) [44] -Inaccessibility of services (e.g., healthcare facilities [44]	
4.4 Structural	
4.4.1 Barriers to risky sexual behavior reduction among youth -Economic constrains [32, 43] -Poverty [32] -Unemployment [32] -Limited economic opportunities [44] -Women’s subordinate status [44] -Cost of services (e.g. secondary education) [39] -Inaccessibility of services (e.g. schools) [39] -Gender-based violence [39]	
4.4.2 Other barriers to intervention success -Poverty [44] -Limited demand for services (e.g., condoms) [44] -Cost of services (e.g., condoms) [44] Total barriers: 42	
5. Characteristics of the strategy of facilitating implementation	5. Characteristics of the strategy of facilitating implementation
5.1 Barriers to intervention acceptability among youth -Use of non-participatory facilitating methods [32]	5.1 Facilitators to intervention acceptability among youth -Use of same sex youth group [36, 37] -Use of different or mixed facilitating methods [35] -Implementation of intervention with fidelity [37]
5.2 Other barriers to intervention success -Failure to implement the intervention with fidelity [44] -Use of non-participatory facilitating methods [44] -Use of corporal punishment [44] -Sexual abuse [44] Total barriers: 5	5.2 Facilitators to youth’s participation in interventions -Mobilization of community members to influence youth to attend the intervention [32] -Integration of intervention with other services [32] -Provision of detailed intervention information to parents [32] -Using outreach activities [32] -Building of a trusting relationship with young people [32] -Use of same age or peer implementers [32] -Provision of incentives [32]
	5.3 Other facilitators to intervention success -Dissemination of intervention information to community members [32, 36, 37] -Implementation of intervention with fidelity [40, 44] -Use of participatory facilitating methods [44] -Decreased corporal punishment [44] -Collaboration among different stakeholders in delivering the intervention [32] Total facilitators: 15
Grand total barriers: 81	Grand total facilitators: 46

### Barriers to HIV prevention interventions for reducing risky sexual behavior among youth

A total of 81 barriers to HIV prevention interventions for reducing risky sexual behavior among youth were identified from the included studies: 42 in the characteristics of the context, 23 in the characteristics of the end users, seven in the characteristics of the implementation object, five in the characteristics of the strategy of facilitating implementation and four in the characteristics of the users domains.

#### Characteristics of the context

Barriers within the characteristics of the context domain were reported by 10 studies [32–34, 36–39, 42–44]. Four types of barriers emerged: interpersonal, community, organizational or institutional and structural barriers. The most common interpersonal barriers identified involve those linked to the reduction of risky sexual behavior among youth, such as partner’s refusal to use an HIV prevention method (e.g., condom use) [32, 33, 38, 39, 42], lack of financial support from family [38, 39, 43], controlling partner [42, 43], peer pressure [38, 43, 44] and lack of child-parent communication on sexual issues [36, 38]. Community barriers that occurred more prominently involve those associated with the reduction of risky sexual behavior among youth. These include gender-biased norms [36, 38, 39, 43, 44], norms discouraging discussion of sexual issues between parents and children [32, 44] and limited resources or services in the community (e.g., programs targeting young people) [32, 33]. Another common set of community barriers identified consists of those associated with young people’s participation in the intervention, such as community violence [34]. Organizational barriers identified involve those linked to the reduction of risky sexual behavior among youth. These include lack of resources (e.g., condoms), inaccessibility of services (e.g., healthcare facilities) and poor quality of services (e.g., lack of confidentiality) [44]. Other organizational barriers reported comprise of those associated with the participation of youth in the intervention. This includes inaccessibility of intervention venue [34, 37]. The most common set of structural barriers reported involve those correlated with the reduction of risky behavior among young people, such as economic constrains [32, 43], gender-based violence [39], unemployment [32] and limited economic opportunities [44].

‘“Safer sex would be safe of course…but at times we can fail to agree on it [condom use]. Eeh… he gives you all sorts of excuses; he is not interested….”’ [38]

‘For the rare young people who may have considered using condoms, access was limited, especially for girls, with intermittent supplies, distant health facilities, and limited confidentiality’ [44]

#### Characteristics of the end users

Seven studies [32, 33, 38, 41–44] identified barriers at the level of characteristics of the end users. Barriers that appeared more prominently include those associated with the reduction of risky sexual behavior among youth. Such barriers include low perceptions of the risk of sexually transmitted infections including HIV [33, 38, 42–44], fear of relationship breakdown [33, 38, 42, 43], being stubborn or hardheaded and desire for pregnancy or children [42, 43]. Another common set of barriers consists of those related to young people’s participation in the intervention, such as concern for privacy [32, 44] and fear of stigma [44].

‘Unprotected sex was also related to factors such as…fear of partner rejection if they suggested condoms…’ [33]

‘“Inviting those with high-risk behaviours to attend HIV sessions raises among them the fear of disclosing their identities to the public and the police.”’ [32]

#### Characteristics of the implementation object

Six studies [32–35, 43, 44] mentioned barriers within the domain of the characteristics of the implementation object. The most common set of barriers identified involves those associated with intervention acceptability among young people. Such barriers include incompatibility of intervention content with the needs of youth [33, 43], long duration of the intervention [33, 35] and complexity of the intervention [35]. Similarly, incompatibility of the intervention content with the needs of community members was mentioned as a barrier to intervention acceptability among community members [44]. Additionally, barriers reported concern those linked to youth’s participation in the intervention, such as restricted days and times of the intervention [34] and age requirements that excluded other youth [33]. Additional barriers that emerged include those associated with the reduction of risky sexual behavior among young people. This includes limited intervention content (e.g., intervention content addresses individual factors such as knowledge without addressing structural factors such as poverty and unemployment) [32, 44].

‘One criticism of the MkV [MEMA kwa Vijana] sessions from some male participants was that they were not linked closely enough to the long-term aspirations of the students.’ [43]

‘…one CBO [community-based organization] staff noted that the upper age limitation of the Link Up project (up to 24 years) likely resulted in lower uptake of services of YMSM [young men who have sex with men] who were partners of MSM [men who have sex with men].’ [33]

‘“….The majority unchanged because education is not enough; they are also in need for other programmes addressing poverty, unemployment and to occupy their spare time.”’ [32]

#### Characteristics of the strategy of facilitating implementation

Two studies [32, 44] mentioned barriers within the characteristics of the strategy of facilitating implementation domain. The category of barriers identified involves those associated with intervention acceptability among youth. This includes the use of non-participatory facilitating methods [32]. Additional barriers to HIV prevention programs for youth reported within this domain include failure to implement the intervention with fidelity, use of corporal punishment and sexual abuse [44].

‘“At the beginning I liked it [program], but later I found it somewhat boring because they continued advising and advising all the time!”’ [32]

#### Characteristics of the users

At the level of characteristics of the users, barriers were reported by two studies [37, 44]. Barriers reported comprise of those linked to intervention acceptability among young people. This includes adult or older implementers [37]. Other barriers to program success identified at this level include implementers’ lack of knowledge related to the content of the intervention, poor education or training of implementers and implementers’ lack of exemplary or positive behavior [44].

‘A few expressed concerns that some facilitators being older might not be able to understand

their experiences.’ [37]

### Facilitators to HIV prevention interventions for reducing risky sexual behavior among youth

There were 46 facilitators to HIV prevention interventions for reducing risky sexual behavior among youth identified from the included studies: 15 in the characteristics of the strategy of facilitating implementation, 13 in the characteristics of the end users, 12 in the characteristics of the context, four in the characteristics of the users and two in the characteristics of the implementation object domains.

#### Characteristics of the strategy of facilitating implementation

Facilitators within this domain were mentioned by six studies [32, 35–37, 40, 44]. Identified facilitators include those linked to intervention acceptability among youth, such as use of same-sex youth group [36, 37], implementation of intervention with fidelity [37] and use of different or mixed facilitating methods [35]. Further facilitators reported involve those correlated with young people’s participation in the intervention, such as provision of incentives, building of a trusting relationship, mobilization of community members to influence youth to attend the intervention, integration of intervention with other services and use of same-age or peer implementers [32]. Other facilitators within this domain include dissemination of intervention information to community members [32, 36, 37], use of participatory facilitating methods, decreased corporal punishment [44] and collaboration among different stakeholders in delivering the intervention [32].

‘They also appreciated the single-sex peer group structure as they felt shy talking about these issues in mixed groups.’ [36]

‘“FSW [female sex workers] sometimes refuse to participate unless paying to them at least YR [Yemen rial] 1000 as a compensation for interrupting their work.”’ [32]

#### Characteristics of the end users

Facilitators within the characteristics of the end users domain were reported by seven studies [32, 36, 37, 39, 42–44]. Facilitators that emerged more prominently consist of those related to the reduction of risky sexual behavior among youth. Such facilitators include fear of pregnancy or sexually transmitted infections including HIV [32, 37, 43], having strong ambitions or being future-oriented [39, 44], having good problem-solving skills, having high self-confidence, being knowledgeable [42], having high self-motivation [39] and low socio-economic status (e.g., lack of money to pay for sex) [44]. Another group of facilitators identified comprises of those associated with young people’s participation in the intervention. This includes perceived benefits of the intervention [37].

‘“…He asked why I wanted him to use a condom and I pretended I was tired of giving birth but in reality, I feared AIDS [acquired immunodeficiency syndrome] as well as pregnancy.”’ [43]

‘One girl explained that the money was not as important as the knowledge she had acquired, and others discussed enjoyment from attending and participating in activities.’ [37]

#### Characteristics of the context

Six studies [33, 38, 39, 42–44] identified facilitators at the characteristics of the context level. Three classifications of facilitators occurred: interpersonal, community and organizational facilitators. The most common interpersonal facilitators reported involve those associated with the reduction of risky sexual behavior among youth. These include partner’s consent to use an HIV prevention method (e.g., condom use) [42, 43], restrictive parenting [38, 44], family support [39, 44], positive peer influence [38] and teacher advice [38]. Similarly, community facilitators that emerged more prominently comprise of those linked to the reduction of risky sexual behavior among young people. Such facilitators include norms encouraging healthy sexual practices (e.g., abstinence and delaying of sexual debut) [44] and religious beliefs discouraging risky sexual behavior [38]. Organizational facilitators reported involve those associated with intervention acceptability among youth. This includes accessibility and friendliness of intervention venue [33].

‘Four girls with reduced risk described receiving significant support from their households specifically to continue with their education.’ [39]

‘“…I can [abstain] because…it also goes against my Christian values to engage in sex before marriage….”’ [38]

‘“…the drop-in-center is a great space, near to the clinic, friendly, freely and wonderful place…”’ [33]

#### Characteristics of the users

Four studies [32, 33, 37, 44] reported facilitators at the characteristics of the users level. Identified facilitators involve those linked to intervention acceptability among youth, such as approachability or friendliness of implementers [33, 37] and experience of implementers [37]. Additional facilitators include training of implementers [32] and implementers’ knowledge of intervention content [44].

‘“…If we are interested in blood test [HIV test], peer educators accompanied us to the clinic, that’s the point I like most…feeling like we are not alone…’” [33]

#### Characteristics of the implementation object

Facilitators within the characteristics of the implementation object domain were identified by four studies [32, 35–37]. Two groups of facilitators emerged: those associated with intervention acceptability among youth and those associated with intervention acceptability among implementers. Compatibility of intervention content with the needs of youth [35–37] was mentioned as a facilitator to intervention acceptability among youth. Relative advantage of the intervention was reported as a facilitator to intervention acceptability among implementers [32].

‘Participants most frequently referenced the KIU [Keep It Up]! content when explaining their reasons for liking the intervention.’ [35]

‘People feel more free, interact more, and ask more sensitive questions in outdoor activities.’ [32]

### Comparison of the identified barriers and facilitators between low\middle- and high-income countries, and male and female youth

There were similarities and differences in identified barriers and facilitators between low/middle- and high-income countries. A major difference noted is that most of the reported barriers within the characteristics of the context domain (mainly community, organizational and structural barriers) affect mostly low/middle-income countries than high-income countries (Additional file 3: Table S1). Also, similarities and differences were observed in reported barriers and facilitators between male and female youth. An important difference observed is that most of the identified barriers and facilitators within the characteristics of the end users and characteristics of the context domains (barriers and facilitators associated with the reduction of risky sexual behavior among youth) affect mostly female youth than male youth. (Additional file 3: Table S2).

## Discussion

This review identified and synthesized barriers and facilitators to HIV prevention interventions for reducing risky sexual behavior among youth (15–24 years) worldwide based on quantitative, qualitative and mixed methods studies published in the last decade. Overall, the findings indicate that barriers and facilitators of HIV prevention intervention for reducing risky sexual behavior among youth comprise of factors associated with the intervention, implementers of the intervention, recipients of the intervention, context of the intervention and strategy of implementing the intervention. Additionally, the most barriers to interventions targeting young people involve factors associated with the context of the intervention and recipients of the intervention. Most facilitators include factors associated with the strategy of implementing the intervention and recipients of the intervention. Other important facilitators consist of factors linked to the context of the intervention.

Similarities exist between this review’s findings when comparing with existing literature. For example, Michielsen et al. 2010 [22] also reported barriers associated with the intervention (e.g., limited intervention content). Barriers linked to the implementers of the intervention (e.g., adult implementers) were also identified in other reviews [21, 25]. Additionally, barriers related to the context of the intervention (e.g., limited resources) were also found in previous reviews [19, 22]. The findings of this review concur with other reviews [22, 23] that identified barriers related to the strategy of delivering the intervention (e.g., failure to implement the intervention with fidelity). Furthermore, this review’s findings are consistent with other reviews [14, 16] that found facilitators associated with the implementers of the intervention (e.g., knowledge and experience of implementers). Other reviews [20, 23, 24] also identified facilitators correlated with the strategy of implementing the intervention (e.g., implementation of intervention with fidelity). Another review [46] also mentioned attributes of recipients of the intervention as factors determining the success of interventions targeting young people.

This review’s findings, however, differ from previous reviews in that the present review found numerous barriers linked to the recipients of the intervention (e.g., fear of relationship breakdown, lack of self-confidence, poor decision-making skills) that were not reported in previous reviews [16, 19–25]. Furthermore, whilst other reviews [16] identified few facilitators associated with the recipients of the intervention (e.g., gender, age and race), the current review found additional facilitators associated with the recipients of the intervention (e.g., fear of pregnancy or sexually transmitted infections, having high self-confidence and having good problem skills). Moreover, previous reviews [16] identified few facilitators related to the context of the intervention, mainly organizational factors (e.g., supportive implementation climate), the present review identified additional facilitators associated with the context of the intervention, mainly interpersonal factors (e.g., positive peer influence, parental advice, family support), community factors (e.g., norms encouraging healthy sexual behaviors and religious beliefs discouraging risky sexual behavior) and organizational factors (e.g., accessibility and friendliness of intervention venue). Additionally, the current review reported additional barriers associated with the strategy of implementing the intervention (e.g., use of non-participatory facilitating methods, use of corporal punishment and sexual abuse) and additional facilitators associated with the strategy of implementing the intervention (e.g., use of participatory facilitating methods, decreased corporal punishment, use of same-age implementers and provision of incentives) not reported in previous reviews [16, 19–25]. Moreover, the present review identified further facilitators linked to the intervention (e.g., compatibility of the intervention with the needs of youth and relative advantage of the intervention) which were not reported in previous reviews [16, 20, 23–25]. The discordance in findings with other reviews may be due to the inclusion of qualitative evidence and the use of a theoretical framework for analysis, which may have contributed to a more holistic findings.

Several limitations should be considered when interpreting the findings of this review. Few included studies (n = 13) in this review could affect the generalizability of the findings. Another limitation is the lack of eligible studies from different high-income countries; all the identified studies (n = 6) from a high-income setting were conducted in the United States of America, and therefore, not representative of all high-income contexts. Additionally, this review was limited to only original peer-reviewed studies; written in English language; and published between January 2010 and April 2022, which may result in publication bias. Also, as some of the included studies did not report the age of youth participants, the review might have included youth not aged 15–24 years. Furthermore, majority of the studies included in this review collected data through self-reports, which may have compromised the quality of the evidence as self-reports are prone to both over-and under-reporting [47]. Whitehead, 2016 [48] argue that the approach to data collection is highly likely to influence the nature and quality of data. Another reason for concern is that determinant frameworks such as the one used to synthesize the factors in this review do not examine causal relationships between the barriers or facilitators and outcomes [17], it is, therefore, difficult to determine with greater certainty whether or not the identified factors influence intervention success.

The review, on the other hand, had its strengths. To the authors’ knowledge, this is the first systematic review incorporating different types of studies to synthesize existing global evidence on barriers and facilitators to HIV prevention interventions for reducing risky sexual behavior among youth. The use of a mixed methods approach most likely results in a more comprehensive understanding of the topic under study [28]. Moreover, the review provides a recent summary of literature from 2010 to 2022. Another strength of this review is the use of a theoretical framework to synthesize the factors. This may have helped to highlight key barriers and facilitators, identify gaps in the literature and formulate theory-driven strategies to facilitate improvements. To the authors’ knowledge, this is the first review to undertake a study of this nature using a theory. Using theory to examine barriers and facilitators adds to the growing body of evidence for the utility of theory to synthesize the factors. Additional strength is that other important population groups in HIV prevention interventions for young people such as implementers, community and family members were represented in this review which may have helped to provide more evidence. Futhermore, most of the included studies (n = 9) were of good quality, and four studies were of fair quality. Therefore, the review findings can reliably inform policies and programmatic strategies to promote the success of HIV prevention interventions for youth.

This systematic review has important implications for practice, policy and research. Considering that the findings of this review suggest that several multi-level barriers and facilitators influence interventions targeting youth, there is need for multi-level approaches to address these factors and enhance intervention success. Multi-level interventions consider barriers and facilitators at multiple levels, evaluate the inter-relationships between these factors, and formulate strategies to enhance intervention success [49]. Evidence suggests that multi-level approaches contribute to the success of an intervention [50]. Furthermore, to promote intervention success, the findings of this review imply the need for combination approaches. Research has established that interventions incorporating different prevention strategies are associated with positive outcomes [46, 51, 52].

Furthermore, the findings of the current review suggest that most barriers to interventions targeting youth include factors associated with the context of the intervention and recipients of the intervention. To enhance intervention success, there is need for more intervention efforts to be directed towards removing barriers associated with context of the intervention and recipients of the intervention. Approaches that have been found successful to address barriers linked to context of the intervention include peer education interventions [53], family-based interventions [54], school-based interventions, community-based interventions, health facility-based interventions [55] and structural interventions [56]. Behavioral or individual risk reduction interventions that use behavior change techniques (e.g., condom-use, communication and motivation enhancement, psycho-education and assertiveness skills training) have been found effective to remove barriers associated with recipients of the intervention [16, 57].

In this review, factors associated with the strategy of implementing the intervention were identified as barriers and facilitators to HIV prevention interventions targeting youth. These findings imply the need for intervention implementers to be sensitive to the delivery strategies they use as some implementation methods are counterproductive. Also, there is need for intervention implementers to ensure that interventions are implemented with fidelity or as intended to enhance intervention success. Evidence suggests that implementation of intervention with fidelity promotes intervention success [23, 24].

Other barriers and facilitators to interventions targeting youth found in the present review include factors associated with the intervention. As guided by Sekhon, 2017’s [58] theoretical framework of intervention acceptability, these findings highlight the importance for intervention designers to develop interventions that are compatible with the needs and values of the target population to enhance program success. To identify the needs of the target population and incorporate them into the intervention, it is recommended that preliminary formative research be conducted as it is linked to intervention success [16].

In this review, factors associated with the implementers of the intervention were some of the reported barriers and facilitators to interventions for young people. These results suggest the need to recruit implementers with desirable characteristics, train them, and provide monitoring, support and supervision to promote intervention success. Research has demonstrated that the use of implementers with attractive attributes (e.g., same-age or peer implementers) promotes intervention success [16, 21].

Findings of this review suggest that barriers and facilitators to HIV prevention programs for reducing risky sexual behavior among youth differ by region (low/middle- and high-income countries) and gender of intervention recipients. These results imply the need for intervention strategies that address the specific barriers and facilitators by region and gender of intervention recipients.

Given the small number of studies identified in the current review, further studies of this nature are recommended. As determinant frameworks do not examine causal relationships between barriers or facilitators and outcomes [17], it is recommended that further experimental studies be conducted to identify and determine the most effective, feasible and acceptable strategies to enhance intervention efficacy. Also, it is recommended that future studies utilize theoretical frameworks to highlight the most important factors that influence interventions targeting youth and formulate theory-driven strategies to promote intervention success.

## Conclusion

This systematic review synthesized current global evidence on barriers and facilitators to HIV prevention interventions for reducing risky sexual behavior among youth. The review shows that barriers and facilitators to HIV prevention interventions for reducing risky sexual behavior among youth include factors associated with the intervention, implementers of the intervention, recipients of the intervention, context of the intervention and strategy of implementing the intervention. Furthermore, the review suggests that most barriers to interventions targeting young people comprise of factors associated with the context of the intervention and recipients of the intervention. Most facilitators involve factors linked to the strategy of implementing the intervention and recipients of the intervention. Other important facilitators include factors associated with the context of the intervention. The findings of this review highlight the need for multi-level and combination approaches to remove barriers and facilitate intervention success. Furthermore, the review suggests the need for further research on barriers and facilitators to HIV prevention interventions for reducing risky sexual behavior among young people.

## Supplementary Information

**Additional file 1. Table S1.** Search strategy in MEDLINE. **Table S2. **Critical appraisal results of included quasi-experimental studies (quantitative studies). **Table S3.** Critical appraisal results of included randomized controlled trial (quantitative study). **Table S4.** Critical appraisal results of included qualitative studies and mixed methods study (qualitative component).

**Additional file 2. **Additional tables.

**Additional file 3. Table S1. **Comparison of the identified barriers and facilitators between low/middle- and high-income countries. **Table S2.** Comparison of the identified barriers and facilitators between male and female youth

## Data Availability

Data that support the findings of this study are included in this article and its additional files.

## Abbreviations

AIDS: Acquired Immunodeficiency Syndrome
CBO: Community-based organization
FSW: Female sex worker
HIV: Human Immunodeficiency Virus
JBI: Joanna Briggs Institute
KIU: Keep It up
MkV: MEMA kwa Vijana
MSM: Men who have sex with men
PRISMA: Preferred Reporting Items for Systematic Reviews and Meta-analysis
UNAIDS: Joint United Nations Programme on HIV/AIDS
WHO: World Health Organization
YR: Yemen rial
YMSM: Young men who have sex with men
