# Correction to: barriers and facilitators to HIV prevention interventions for reducing risky sexual behavior among youth worldwide: a systematic review

**DOI:** 10.1186/s12879-022-07762-z

**Published:** 2022-10-11

**Authors:** Fungai Mbengo, Esther Adama, Amanda Towell-Barnard, Arvin Bhana, Maggie Zgambo

**Affiliations:** 1 School of Nursing and Midwifery, Edith Cowan University, 6027 Joondalup, WA Australia; 2 Health Systems Research Unit, South African Medical Research Council, 7505 Tygerberg, South Africa; 3 Centre for Rural Health, School of Nursing and Public Health, University of KwaZulu-Natal, 4041 Durban, South Africa

**Correction to: BMC Infect Dis 22, 679 (2022)**

**https://doi.org/10.1186/s12879-022-07649-z**.

Prior to the publication of this article several changes could not be implemented. These changes are:

## Figure 1

The text:

- Records identified through electronic search: CINAHL, Cochrane Library, Google Scholar, MEDLINE, PsychINFO, ProQuest Central and Web of Science datababes, Cambridge and Oxford journals, UNAIDS and WHO websites (n = 7823)

Should read:

- Records identified through electronic search: Cambridge Core, CINAHL, Cochrane Library, Google Scholar, MEDLINE, ProQuest Central, PsycINFO, Oxford Journals and Web of Science databases, UNAIDS and WHO websites (n = 7823)

## Table 1

The text:

- Author, year & title.

Should read:

- Author & year.

The original publication has been updated to correct these errors.

