# Supplementary material for: Barriers and facilitators to HIV prevention interventions for reducing risky sexual behavior among youth worldwide: a systematic review
Source: BMC Infect Dis. 2022 Aug 8;22:679. doi: 10.1186/s12879-022-07649-z (PMC9361597; doi:10.1186/s12879-022-07649-z)
Supplement: Supplementary file 1 — Additional file 1. Table S1. Search strategy in MEDLINE. Table S2. Critical appraisal results of included quasi-experimental studies (quantitative studies). Table S3. Critical appraisal results of included randomized controlled trial (quantitative study). Table S4. Critical appraisal results of included qualitative studies and mixed methods study (qualitative component). [file 12879_2022_7649_MOESM1_ESM.docx]

**Additional File 1: Table S1: Search strategy in MEDLINE**

| **Number** | **Search terms** |
| --- | --- |
| #1 | Youth [Text Word] OR Adolescen* [Mesh Term] OR “Young people” [Text Word] OR Teen* [Text Word] OR “Young adults” [Text Word] OR Students [Text Word] |
| #2 | Barriers [Text Word] OR Challenges [Text Word] OR Constrains [Text Word] OR Difficulties [Text Word] OR Obstacles [Text Word] OR Issues [Text Word] OR Problems [Text Word] OR Limitations [Text Word] |
| #3 | Facilitators [Text Word] OR Motivators [Text Word] OR Enablers [Text Word] OR “Enabling Factors” [Text Word] OR Contributors [Text Word] OR “Contributing Factors” [Text Word] OR “Promoting Factors” [Text Word] |
| 4# | “HIV prevention intervention” [Text Word] OR “HIV prevention strateg*” [Text Word] OR “HIV prevention program*” [Text Word] OR “HIV education prevention program*”[Text Word] OR “Sexual* education program*” [Text Word] OR “Health behav* intervention” [Text Word] OR “Peer* education program*”[Text Word] OR “Peer* HIV prevention intervention” [Text Word] OR “HIV prevention model” [Text Word] OR “HIV/sexually transmitted infection/pregnancy intervention” [Text Word] OR “HIV/AIDS/STI reduction intervention” [Text Word] OR “HIV/STD risk reduction intervention” [Text Word] OR “Behav* intervention” [Text Word] OR “Structural intervention” OR “Behav* intervention” OR “Combin* intervention” OR “Multicomponent intervention” [Text Word] OR “Sexual health intervention” [Text Word] OR “Life skills intervention” [Text Word] OR “HIV/AIDS prevention intervention” [Text Word] OR “HIV/AIDS prevention program*”[Text Word] OR “Behav* change intervention” [Text Word] OR “Behav* change program* [Text Word] |
| 5# | “Risk* sexual behav*” [Text Word] OR “Unsafe sex” [Text Word] OR “High risk* sex” [Text Word] OR “Unhealthy sexual relations” [Text Word] OR “Sexual health behav*” [Text Word] OR “Sexual risk* behav*” [Text Word] OR “HIV/sexually transmitted infection risk behav*” [Text Word] OR “Sexual risk-taking behav*” [Text Word] OR “Risk* behav* practices” [Text Word] OR “Risk* behav*” [Text Word] OR “Health risk* behav*” [Text Word] OR “Sexual partners” [Text Word] OR “Sexual behav*” [Text Word] OR “Forced sex” [Text Word] OR “Coerced sex” [Text Word] OR “Early sexual debut” [Text Word] OR “Early marriage” [Text Word] OR “Transactional sex” [Text Word] OR “Intergenerational sex” [Text Word] OR Multiple sexual partners [MeSH term] OR “Sex partners” [Text Word] OR “Multiple sex partners” OR “Concurrent sexual partners” [Text Word] OR “Condomless sex” [Text Word] OR “Unprotected sex” [Text Word] OR “Inconsistent condom use” [Text Word] OR “Pregnancy incidence” [Text Word] |
| 6# | Eff* [Text Word] OR Success [Text Word] OR Outcome [Text Word] OR Impact [Text Word] OR Evaluat* [Text Word] OR Assess* [Text Word] OR Treatment outcome [Mesh Term] OR Program evaluation [Mesh Term] OR “Process evaluation” [Text Word] |
| 7# | Implementation [Text Word] OR Implementation science [Mesh Term] OR “Implementation plan” [Text Word] OR “Implementation methods” [Text Word] OR “Implementation strategies” [Tex Word] OR “Implementation tactics” [Text Word] OR “Implementation approaches” [Text Word] OR Implementing [Text Word] Or Embedding [Text Word] OR Facilitating [Text Word] OR Delivering [Text Word] OR Entrenching [Text Word] |
| #8 | 1 AND 2 AND 3 AND 4 AND 5 AND 6 AND 6 AND 7 = Youth [Text Word] OR Adolescen* [Mesh Term] OR “Young people” [Text Word] OR Teen* [Text Word] OR “Young adults” [Text Word] OR Students [Text Word] AND Barriers [Text Word] OR Challenges [Text Word] OR Constrains [Text Word] OR Difficulties [Text Word] OR Obstacles [Text Word] OR Issues [Text Word] OR Problems [Text Word] OR Limitations [Text Word] AND Facilitators [Text Word] OR Motivators [Text Word] OR Enablers [Text Word] OR “Enabling Factors” [Text Word] OR Contributors [Text Word] OR “Contributing Factors” [Text Word] OR “Promoting Factors” [Text Word] AND “HIV prevention intervention” [Text Word] OR “HIV prevention strateg*” [Text Word] OR “HIV prevention program*” [Text Word] OR “HIV education prevention program*”[Text Word] OR “Sexual* education program*” [Text Word] OR “Health behav* intervention” [Text Word] OR “Peer* education program*”[Text Word] OR “Peer* HIV prevention intervention” [Text Word] OR “HIV prevention model” [Text Word] OR “HIV/sexually transmitted infection/pregnancy intervention” [Text Word] OR “HIV/AIDS/STI reduction intervention” [Text Word] OR “HIV/STD risk reduction intervention” [Text Word] OR “Behav* intervention” [Text Word] OR “Structural intervention” OR “Behav* intervention” OR “Combin* intervention” OR “Multicomponent intervention” [Text Word] OR “Sexual health intervention” [Text Word] OR “Life skills intervention” [Text Word] OR “HIV/AIDS prevention intervention” [Text Word] OR “HIV/AIDS prevention program*”[Text Word] OR “Behav* change intervention” [Text Word] OR “Behav* change program* [Text Word] AND “Risk* sexual behav*” [Text Word] OR “Unsafe sex” [Text Word] OR “High risk* sex” [Text Word] OR “Unhealthy sexual relations” [Text Word] OR “Sexual health behav*” [Text Word] OR “Sexual risk* behav*” [Text Word] OR “HIV/sexually transmitted infection risk behav*” [Text Word] OR “Sexual risk-taking behav*” [Text Word] OR “Risk* behav* practices” [Text Word] OR “Risk* behav*” [Text Word] OR “Health risk* behav*” [Text Word] OR “Sexual partners” [Text Word] OR “Sexual behav*” [Text Word] OR “Forced sex” [Text Word] OR “Coerced sex” [Text Word] OR “Early sexual debut” [Text Word] OR “Early marriage” [Text Word] OR “Transactional sex” [Text Word] OR “Intergenerational sex” [Text Word] OR Multiple sexual partners [MeSH term] OR “Sex partners” [Text Word] OR “Multiple sex partners” OR “Concurrent sexual partners” [Text Word] OR “Condomless sex” [Text Word] OR “Unprotected sex” [Text Word] OR “Inconsistent condom use” [Text Word] OR “Pregnancy incidence” [Text Word] AND Eff* [Text Word] OR Success [Text Word] OR Outcome [Text Word] OR Impact [Text Word] OR Evaluat* [Text Word] OR Assess* [Text Word] OR Treatment outcome [Mesh Term] OR Program evaluation [Mesh Term] OR “Process evaluation” [Text Word] AND Implementation [Text Word] OR Implementation science [Mesh Term] OR “Implementation plan” [Text Word] OR “Implementation methods” [Text Word] OR “Implementation strategies” [Tex Word] OR “Implementation tactics” [Text Word] OR “Implementation approaches” [Text Word] OR Implementing [Text Word] Or Embedding [Text Word] OR Facilitating [Text Word] OR Delivering [Text Word] OR Entrenching [Text Word] |
| **9# Limits** | Publications from 2010/01/01 to 2022/04/05  Age outside 15 – 24 years  Non-English publications |

**Additional File 1: Table S2: Critical appraisal results of included quasi-experimental studies (quantitative studies)**

| **Study** | **Q1** | **Q2** | **Q3** | **Q4** | **Q5** | **Q6** | **Q7** | **Q8** | **Q9** | **Total % per paper** |
| --- | --- | --- | --- | --- | --- | --- | --- | --- | --- | --- |
| Garofalo et al., 2012 | Y | Y | Y | N | Y | Y | Y | Y | Y | 89 |
| Greene et al., 2016 | Y | Y | Y | N | Y | Y | Y | Y | Y | 89 |
| Rohrbach et al., 2019 | Y | Y | Y | Y | Y | N | Y | Y | Y | 89 |
| **Total % per question** | 100 | 100 | 100 | 33 | 100 | 67 | 100 | 100 | 100 |  |

Y = Yes, N = No, U = Unclear, NA = Not Applicable; JBI critical appraisal checklist for quasi-experimental studies: Q1 = Is it clear in the study what is the ‘cause’ and what is the ‘effect’ (i.e. there is no confusion about which variable comes first)?; Q2 = Were the participants included in any comparisons similar?; Q3 = Were the participants included in any comparisons receiving similar treatment/care, other than the exposure or intervention of interest?; Q4 = Was there a control group?; Q5 = Were there multiple measurements of the outcome both pre and post the intervention/exposure?; Q6 = Was follow up complete and if not, were differences between groups in terms of their follow up adequately described and analyzed?; Q7 = Were the outcomes of participants included in any comparisons measured in the same way?; Q8 = Were outcomes measured in a reliable way?; Q9 = Was appropriate statistical analysis used?

**Additional File 1: Table S3: Critical appraisal results of included randomized controlled trial (quantitative study)**

| **Study** | **Q1** | **Q2** | **Q3** | **Q4** | **Q5** | **Q6** | **Q7** | **Q8** | **Q9** | **Q10** | **Q11** | **Q12** | **Q13** | **Total % per paper** |
| --- | --- | --- | --- | --- | --- | --- | --- | --- | --- | --- | --- | --- | --- | --- |
| Sales et al., 2012 | Y | Y | Y | Y | U | U | Y | Y | Y | Y | Y | Y | Y | 85 |
| **Total % per question** | 100 | 100 | 100 | 100 | 0 | 0 | 100 | 100 | 100 | 100 | 100 | 100 | 100 |  |

Y = Yes, N = No, U = Unclear, NA = Not Applicable; JBI critical appraisal checklist for randomized controlled trials: Q1 = Was true randomization used for assignment of participants to treatment groups?; Q2 = Was allocation to treatment groups concealed?; Q3 = Were treatment groups similar at baseline?; Q4 = Were participants blind to treatment assignment?; Q5 = Were those delivering treatment blind to treatment assignment?; Q6 = Were outcome assessors blind to treatment assignment?; Q7 = Were treatment groups treated identically other than the intervention of interest?; Q8 = Was follow-up complete, and if not, were strategies to address incomplete follow-up utilized?; Q9 = Were participants analyzed in the groups to which they were randomized?; Q10 = Were outcomes measured in the same way for treatment groups?; Q11 = Were outcomes measured in a reliable way?; Q12 = Was appropriate statistical analysis used?; Q13 = Was the trial design appropriate, and any deviations from the standard RCT design (individual randomization, parallel groups) accounted for in the conduct and analysis of the trial?

**Additional File 1: Table S4: Critical appraisal results of included qualitative studies and mixed methods study (qualitative component)**

| **Study** | **Q1** | **Q2** | **Q3** | **Q4** | **Q5** | **Q6** | **Q7** | **Q8** | **Q9** | **Q10** | **Total % per paper** |
| --- | --- | --- | --- | --- | --- | --- | --- | --- | --- | --- | --- |
| Al-iryani et al., 2011 | Y | Y | Y | Y | Y | N | N | Y | Y | Y | 80 |
| Aung et al., 2017 | U | Y | Y | Y | Y | N | N | Y | Y | Y | 70 |
| Jewkes et al., 2010 | N | Y | Y | Y | Y | N | N | Y | Y | Y | 70 |
| Morrison-Beedy et al., 2013 | N | Y | Y | Y | Y | N | N | Y | Y | Y | 70 |
| Musiimenta., 2012 | N | Y | Y | Y | Y | N | N | Y | Y | Y | 70 |
| Ridgeway et al., 2020 | Y | Y | Y | Y | Y | N | Y | Y | Y | Y | 90 |
| Sales et al., 2012a | Y | Y | Y | Y | Y | Y | Y | Y | Y | Y | 100 |
| Wamoyi et al., 2012 | U | Y | Y | Y | Y | Y | N | Y | Y | Y | 80 |
| Wight et al., 2012 | Y | Y | Y | Y | Y | N | N | Y | Y | Y | 80 |
| **Total %** | 44 | 100 | 100 | 100 | 100 | 22 | 22 | 100 | 100 | 100 |  |

Y = Yes, N = No, U = Unclear, NA = Not Applicable; JBI critical appraisal checklist for qualitative research: Q1 = Is there congruity between the stated philosophical perspective and the research methodology?; Q2 = Is there congruity between the research methodology and the research question or objectives?; Q3 = Is there congruity between the research methodology and the methods used to collect data?; Q4 = Is there congruity between the research methodology and the representation and analysis of data?; Q5 = Is there congruity between the research methodology and the interpretation of results?; Q6 = Is there a statement locating the researcher culturally or theoretically?; Q7 = Is the influence of the researcher on the research, and vice- versa, addressed?; Q8 = Are participants, and their voices, adequately represented?; Q9 = Is the research ethical according to current criteria or, for recent studies, and is there evidence of ethical approval by an appropriate body?; Q10 = Do the conclusions drawn in the research report flow from the analysis, or interpretation, of the data?
