# Supplementary material for: Barriers and facilitators to HIV prevention interventions for reducing risky sexual behavior among youth worldwide: a systematic review
Source: BMC Infect Dis. 2022 Aug 8;22:679. doi: 10.1186/s12879-022-07649-z (PMC9361597; doi:10.1186/s12879-022-07649-z)
Supplement: Supplementary file 2 — Additional file 2. Additional tables. [file 12879_2022_7649_MOESM2_ESM.docx]

**Additional file 2: Table S1:** **Joanna Briggs Institute’s degree of evidence for findings of included qualitative studies**

| **Degree of evidence** | **Interpretation** |
| --- | --- |
| Unequivocal | Evidence beyond a reasonable doubt that may include findings that are a matter of fact, directly reported/observed and not open to challenge |
| Plausible/credible | Notwithstanding an interpretation, plausible in light of the data and theoretical framework. The interpretations can be logically inferred from the data but, because the findings are inherently interpretive, they can be challenged. |
| Unsupported | When neither of the other level descriptors applies and when, most notably, findings are not supported by the data |

**Additional file 2: Table S2: Data extraction from Al-iryani et al., 2011. Process evaluation of a three-year community-based peer education intervention for HIV prevention among Yemeni young people**

| **Findings** | **Illustration** | **Evidence** | | |
| --- | --- | --- | --- | --- |
|  |  |  | | |
|  |  | **Unequivocal** | **Credible** | **Unsupported** |
| F1. Collaboration among different stakeholders in delivering the intervention promoted awareness among community members | ‘Raising awareness among community members is the result of the cooperation between local council members, civil society organizations, and community-based organizations—especially the Social Service Centre and its leadership.’ (local council member). P. 143. | Unequivocal |  |  |
| F2. Collaboration among implementers in delivering the intervention enhanced intervention success | The main role of the community focal points was to facilitate the role of the peer educators by mobilizing young people to attend the peer education sessions. P. 144. |  | Credible |  |
| F3. Mobilization of community members to influence youth to attend the intervention facilitated youth’s participation in the intervention | ‘We approach parents, head of the neighbourhoods, the close friends of the targeted youth to influence them.’ (male community focal point). P. 144. | Unequivocal |  |  |
| F4. Integration of intervention with other services promoted youth’s participation in the intervention | ‘At the beginning, people in Sesaban did not welcome me, but after birth certificates and food ration services, their attitudes had been totally changed.’ (female community focal point). P 144. | Unequivocal |  |  |
| F5. Provision of detailed intervention information to parents encouraged youth’s participation in the intervention | Even when they refuse in the beginning, older female community focal points pay another visit to explain the objective of the sessions. Also, community focal points always show their ID badge and an official letter from the CBO with details on the peer education timing and venue. P. 145. |  | Credible |  |
| F6. Training of implementers enhanced success of the intervention | Local council members also stated that having well trained peer educators who can communicate the information to people with various educational levels had played a big role in the success of the intervention. P. 145. |  | Credible |  |
| F7. Training of implementers enhanced success of the intervention | They said that the life-skills they acquired…. had enabled them to efficiently pursue their roles as community focal points and mobilize young people to participate in peer education activities. P. 146. |  | Credible |  |
| F8. Use of outreach activities facilitated youth’s participation in the intervention | ‘Addressing people in their usual settings helps us to avoid many problems when trying to recruit participants.’ (male peer educator). P. 146. | Unequivocal |  |  |
| F9. Relative advantage of the intervention (outdoor activities) promoted intervention acceptability among implementers | ‘People feel more free, interact more, and ask more sensitive questions in outdoor activities.’ (female community focal point). P. 146. | Unequivocal |  |  |
| F10. Relative advantage of intervention (outdoor peer education) facilitated intervention acceptability among implementers | Another benefit of outdoor peer education, as stated by peer educators and community focal points, is that the time of sessions, which are usually conducted between 3:00 pm and 6:00 pm, is Qat chewing time, and many young people are not interested to give up chewing, since chewing is not allowed in CBOs and CSOs. P. 146-7. |  | Credible |  |
| F11. Relative advantage of the intervention (outreach peer education) enabled intervention acceptability among implementers | ‘Outreach peer education is a preferable education modality.’ (local council member). P. 147. | Unequivocal |  |  |
| F12. Use of non-participatory facilitating methods impeded intervention acceptability female among youth | ‘At the beginning I liked it, but later I found it somewhat boring because they continued advising and advising all the time!’ (young woman from Basateen). P. 147. | Unequivocal |  |  |
| F13. Dissemination of intervention information to community members enhanced awareness of the program | ‘……I have started educating my neighbor.’ (targeted young woman from Memdarah). P. 147. | Unequivocal |  |  |
| F14. Myths about contraceptives deterred risky sexual behavior reduction among female youth | ‘Some have the myth that two condoms are needed to provide protection against HIV transmission.’ (young woman from Memdarah). P. 147. | Unequivocal |  |  |
| F15. Norms discouraging discussion of sexual issues between parents and children impeded risky sexual behavior reduction among female youth | ‘Some girls feel shy and many families found it unsuitable to speak about condoms.’ (young woman from Sesaban). P. 148 | Unequivocal |  |  |
| F16. Fear of sexually transmitted infections including HIV facilitated risky sexual behavior reduction among youth | ‘I asked my husband to use the condom because he once got a bloody inflammation. I’m not sure about his faithfulness.’ (young woman from Abdul Qaui). | Unequivocal |  |  |
| F17. Partner’s refusal of HIV testing hindered risky sexual behavior reduction among female youth | ‘I’m now engaged. Despite that my fiancé is good, I’m insisting that he should test for AIDS but he is still refusing.’ (young woman from Memdarah). P. 148. | Unequivocal |  |  |
| F18. Parent’s refusal of HIV testing impeded risky sexual behavior reduction among youth | ‘I want to be sure he is free of AIDS. Even, I want myself to test for AIDS but my mother refused…...’ (young woman from Memdarah). P. 148. | Unequivocal |  |  |
| F19. Fear of AIDS (HIV) promoted risky sexual behavior reduction among youth | ‘I decided to use condoms after my friend died of AIDS.’ (a 24-year-old FSW. P. 148. | Unequivocal |  |  |
| F20. Negative attitudes towards condom use deterred risky sexual behavior reduction among female youth | ‘I don’t use condoms because they always get torn and men feel more pleasure without them. . . . I also feel more pleasure without them.’ (23-year-old FSW). P. 149. | Unequivocal |  |  |
| F21. Building of a trusting relationship with young people promoted female youth’s participation in the intervention | Building a trust relationship with FSW was the starting point to have them participate in peer education sessions, as they were always concerned to be caught by the police. P. 149 |  | Credible |  |
| F22. Concern for privacy hindered female youth’s participation in the intervention | ‘Inviting those with high-risk behaviours to attend HIV sessions raises among them the fear of disclosing their identities to the public and the police.’ (female community focal point). P. 149. | Unequivocal |  |  |
| F23. Provision of incentives encouraged female youth’s participation in the intervention | ‘FSW sometimes refuse to participate unless paying to them at least YR 1000 as a compensation for interrupting their work.’ (female community focal point). P. 149. | Unequivocal |  |  |
| F24. Use of same age implementers promoted female youth’s participation in the intervention | ‘Once I was unable to target the famous gatekeeper who brings girls to the hotels, but I managed that by contacting a younger gatekeeper who facilitated our meeting to educate FSW who worked with that famous pimp.’ (female community focal point) | Unequivocal |  |  |
| F25. Poverty, unemployment, economic constrains and limited resources/programs deterred risky sexual behavior reduction among youth | Poverty, unemployment, economic hardship, and lack of programs addressing youth in communities was mentioned by peer educators as underlying causes for not having an environment inductive for behavioral change among FSWs. P. 149. |  | Credible |  |
| F26. Inadequate intervention content (intervention content addresses individual-level factors (e.g., knowledge) without addressing structural factors (e.g., poverty, unemployment) deterred risky sexual behavior reduction among female youth | ‘….The majority unchanged because education is not enough; they are also in need for other programmes addressing poverty, unemployment and to occupy their spare time.’(female peer educator). P.149. | Unequivocal |  |  |

**Additional file 2: Table S3:** **Data extraction from Aung et al., 2017. Effectiveness of an integrated community-and clinic-based intervention on HIV testing, HIV knowledge, and sexual risk behavior of young men who have sex with men (YMSM) in Myanmar**

| **Findings** | **Illustration** | **Evidence** | | |
| --- | --- | --- | --- | --- |
|  |  | **Unequivocal** | **Credible** | **Unsupported** |
| F27. Approachability/friendliness of implementers facilitated intervention acceptability among youth. | ‘…If we ask them a question, they explained us patiently. How lovely they are!’ (one 22-year-old YMSM participant). P. S49. | Unequivocal |  |  |
| F28. Accessibility and friendliness of the intervention venue promoted intervention acceptability among youth. | ‘…the drop-in-center is a great space, near to the clinic, friendly, freely and wonderful place….’ (21-year-old YMSM participant). P. S49. | Unequivocal |  |  |
| F29. Approachability/friendliness of implementers enabled intervention acceptability among youth. | . ‘…If we are interested in blood test [HIV test], peer educators accompanied us to the clinic, that’s the point I like most..feeling like we are not alone….’ (another YMSM participant aged 19 years). P. S49. | Unequivocal |  |  |
| F30. Age requirements that excluded other youth deterred youth’s participation in the intervention. | …one CBO staff noted that the upper age limitation of the Link Up project (up to 24 years) likely resulted in lower uptake of services of YMSM who were partners of MSM. P. S49. |  | Credible |  |
| F31. Incompatibility of intervention content with the needs of youth impeded intervention acceptability among youth. | Another CBO staff mentioned that some YMSM preferred to have a “one-stop shop” where they get testing and treatment done in the same place as opposed to needing a referral for HIV treatment. P. S49. |  | Credible |  |
| F32. Long duration of the intervention hindered intervention acceptability among youth. | ‘…the clients prefer quick process…they don’t want to stay at the clinic longer duration…’ (a peer educator). P. S49. | Unequivocal |  |  |
| F33. Concern for privacy deterred risky sexual behavior reduction among youth | Participants commented on constraints in possessing condoms, and fearing friends or family might discover them. P. 550. |  | Credible |  |
| F34. Fear of relationship breakdown, low perception of risk of sexually transmitted infections including HIV, partner’s refusal of using condoms and limited resources in the community (e.g. condoms) impeded risky sexual behavior reduction among male youth. | Unprotected sex was also related to factors such as partner trust, condom availability, fear of partner rejection if they suggested condoms, and partners’ or clients’ refusal or disapproval of using condoms. P. 550. |  | Credible |  |

**Additional file 2: Table S4: Data extraction from Garofalo et al., 2012. Life skills: evaluation of a theory-driven behavioral HIV prevention intervention for young transgender women**

| **Findings** | **Illustration** |
| --- | --- |
| F35. Restricted days and times of the intervention, inaccessibility of the intervention venue, violence in the community and incarceration hindered youth’s participation in the intervention | However, we did record reasons for non-attendance from these participants; such reasons included scheduling conflicts, incarceration, transportation issues, and suffering assault in the neighborhood (and thus not wanting to return to the neighborhood). P. 428. |

**Additional file 2: Table S5: Data extraction from Greene et al., 2016. Implementation and evaluation of the Keep It Up! online HIV prevention intervention in a community-based setting**

| **Findings** | **Illustration** |
| --- | --- |
| F36.Compatability of intervention content with the needs of youth promoted intervention acceptability among youth | Participants most frequently referenced the KIU! content when explaining their reasons for liking the intervention (168 excerpts). P. 239.  “I found the program extremely helpful because it encounters real situation within the community such as hooking up online and or bars. I think it can be of great help to a young crowd that has not much experience into the gay scene.” P. 239. |
| F37. Using different facilitating methods facilitated intervention acceptability among youth | The format of KIU! was the second most frequently cited reason given by participants for liking the intervention (140 excerpts). P. 239.  “The different array of media used to get the message across was very fun.” P. 239. |
| F38. Compatibility of intervention content with the needs of youth promoted intervention acceptability among youth | “ It got me to just sit down and think about my own practices and own up to some of the risky behavior that I’ve been a part of” and “I loved how real and honest it was. I wish all gay kids had this as their sex education in high school.” (105 excerpts). P. 240. |
| F39. Long duration of the intervention deterred intervention acceptability among youth. | Participants complained that the intervention was too long and slow. P. 241.  “Overall, the program was useful . . .it was kind of longer than I expected . . . But I understand you guys are trying to get the point across.” P.241. |
| F40. Complexity of the intervention impeded intervention acceptability among youth. | Participants who disliked the intervention based on technical problems mentioned Internet browser incompatibility and glitches that caused the intervention to load slowly or freeze. P.241. |

**Additional file 2: Table S6: Data extraction from Jewkes et al., 2010. ‘I woke up after I joined Stepping Stones’: meanings of an HIV behavioural intervention in rural South African young people's lives**

| **Findings** | **Illustration** | **Evidence** | | |
| --- | --- | --- | --- | --- |
|  |  | **Unequivocal** | **Credible** | **Unsupported** |
| F41. Dissemination of intervention information to community members promoted awareness of the program | Loyiso, a 17-year-old women from Mthatha, started advising friends on how to use condoms to protect against HIV and pregnancy. P. 1078. |  | Credible |  |
| F42. Lack of parent-child communication on sexual issues deterred risky sexual behavior reduction among youth | One village girl said her mother stopped her discussing boyfriends by saying that she ‘was too young to have one’. P. 1078. |  | Credible |  |
| F43. Compatibility of intervention content with the needs of youth promoted intervention acceptability among youth | …the programme enabled participants to move beyond such resistance to ‘AIDS messages’ when found they were offered a chance to talk about what they perceived as real issues in their lives, such as relationship problems and sex, and they enjoyed this. P. 1081. |  | Credible |  |
| F44. Use of same sex youth group promoted intervention acceptability among youth | They also appreciated the single-sex peer group structure as they felt shy talking about these issues in mixed groups. P. 1081. |  | Credible |  |
| F45. Gender-biased norms hindered risky sexual behavior reduction among female youth | In this locality, men generally controlled sexual encounters and thus it is unsurprising that they conveyed a clear sense of agency in relation to condom use. In marked contrast, just two or three of the women said they started consistently to use condoms. |  | Credible |  |
| F46. Violent partner deterred risky sexual behavior reduction among female youth | Some had suggested condom use to a partner and been met with hostility and threats of violence. P.1081. |  | Credible |  |
| F47. Gender-biased norms impeded risky sexual behavior reduction among female youth | In a context where concurrent partners were the norm, women had to work hard to be the most desirable. They feared losing their position as a main partner (and in the village, potentially wife) to another woman, or failing to achieve that status by overcoming a rival, if they did not try hard to ensure that sex with them was better than with the other women. P. 1081. |  | Credible |  |
| F48. Intentions/readiness to change promoted risky sexual behavior reduction among female youth | Nonetheless, many of these women did something aimed at reducing their risk of HIV. P. 1082. |  | Credible |  |

**Additional file 2: Table S7: Data extraction from Morrison-Beedy et al., 2013. Exit interviews from adolescent girls who participated in a sexual risk‐reduction intervention: implications for community‐based health education promotion for adolescents**

| **Findings** | **Illustration** | **Evidence** | | |
| --- | --- | --- | --- | --- |
|  |  | **Unequivocal** | **Credible** | **Unsupported** |
| F49. Perceived benefits of the intervention encouraged youth’s participation in the intervention. | One girl explained that the money was not as important as the knowledge she had acquired, and others discussed enjoyment from attending and participating in activities. P. 3. |  | Credible |  |
| F50. Inaccessibility of the intervention venue deterred youth’s participation in the intervention | ‘And that was, my hardest thing about it too is the transportation. There was times where I had to walk from [approximately 2 miles] to here but I still did … it was somethin’ to do.’ (one girl). P. 3. | Unequivocal |  |  |
| F51. Fear of sexually transmitted infections including HIV promoted risky sexual behavior reduction among youth | Several girls in our group reported using condoms even with steady partners due to mistrust and concerns they might be exposed to HIV/STIs. P. 4. |  | Credible |  |
| F52. Compatibility of intervention content with the needs of youth promoted intervention acceptability among youth | ‘There’s not a lot [of] places or people to talk to about stuff like this….’ (one participant). P. 5. | Unequivocal |  |  |
| F53. Use of same sex youth group promoted intervention acceptability among youth | ‘And when you get a bunch of girls together, it…it brings it to the table..’ (one participant). P. 5. | Unequivocal |  |  |
| F54. Approachability  /friendliness of implementers encouraged intervention acceptability among youth | ‘They was nice. They was polite. They wasn’t rude. And they wasn’t attitudy. Because you know some… alotta women have a nasty attitude.’ (one of the girls). P. 5. | Unequivocal |  |  |
| F55. Adult implementers deterred intervention acceptability among youth | A few expressed concerns that some facilitators being older might not be able to understand their experiences. P. 5. |  | Credible |  |
| F56. Experience of implementers promoted intervention acceptability among youth | However, one girl stated that she preferred having older facilitators feeling they delved deeper into topics and helped guide the girls around sensitive issues and concerns. P. 5. |  | Credible |  |
| F57. Adult implementers deterred intervention acceptability among youth | Only one girl expressed her dislike, referring to them as “boring old women”. P. 5. |  | Credible |  |
| F58. Use of same sex youth group promoted intervention acceptability among youth | ‘I think everybody would attend, but they wouldn’t talk and conversate because the boys, well the boys would, but the girls wouldn’t because they’d be so uncomfortable with the boys talking about sex.’ (one girl). P. 5. | Unequivocal |  |  |
| F59. Dissemination of intervention information to community members promoted awareness of the program | Several reported trying to educate their mothers who were unfamiliar with sexual health information. Some participants discussed information with other female adults, such as sisters or grandmothers, who were important to them. P. 6. |  | Credible |  |
| F60. Dissemination of intervention information to community members enhanced awareness of the program | Most reported they had, hoping to educate or recruit friends or family into the ongoing intervention. P. 6 |  | Credible |  |
| F61. Dissemination of intervention information to community members promoted awareness of the program | ‘I took it home and I showed my sisters and stuff. ‘Oh yea we see this stuff in health’ and I was’ like, “yea, well, you need to pay attention to it in health.”’(another girl). P. 6. | Unequivocal |  |  |
| F62. Implementation of the intervention with fidelity | One girl stated that she was shocked by the amount of knowledge she had acquired through the intervention feeling she had not been taught these facts in health classes. P. 6. |  | Credible |  |
| F63. Dissemination of intervention information to community members enabled awareness of the program | …...Some girls reported talking to older brothers and male cousins about what they learned in the intervention. P. 6. |  | Credible |  |
| F64. Dissemination of intervention information to community members promoted awareness of the program | ‘Some of the stuff they taught me at HIPTeens, I was talking to one of the dudes I was messing with and he didn’t know that stuff. And he’s older than me.’ (one girl). P.6. | Unequivocal |  |  |

**Additional file 2: Table S8: Data extraction from Musiimenta., 2012. Contextual mediators influencing the effectiveness of behavioural change interventions: A case of HIV/AIDS prevention behaviours**

| **Findings** | **Illustration** | **Evidence** | | |
| --- | --- | --- | --- | --- |
|  |  | **Unequivocal** | **Credible** | **Unsupported** |
| F65. Partner’s negative attitudes towards condom use impeded reduction of risky sexual behavior among female youth | ‘…but my boy friend calls them paper bags [condoms], and says it is like eating a sweet without removing it from its cover......’ (female youth). P. 8 | Unequivocal |  |  |
| F66. Partner’s refusal of condom use deterred risky sexual behavior reduction among female youth | ‘Safer sex would be safe of course…but at times we can fail to agree on it [condom use]. Eeh… he gives you all sorts of excuses; he is not interested…..’ (female youth). P. 8. | Unequivocal |  |  |
| F67. Fear of relationship breakdown hindered risky sexual behavior reduction among female youth | ‘……and when you over insist you can break your relationship because he may end up leaving me and go in for girls who are willing.’ (female youth). P. 8. | Unequivocal |  |  |
| F68. Low perceptions of risk of HIV infection deterred risky sexual behavior reduction among male youth | ‘…….We have loved each other for a long time, and we know we don't have AIDS , we really don’t need to use condoms.’ (male youth). P. 8. | Unequivocal |  |  |
| F69. Lack of parent-child communication on sexual issues impeded risky sexual behavior reduction among male youth | ‘Parents should be open to us in time and tell us to abstain or how protect ourselves from AIDS and pregnancy. Mine weren’t [open]…there are certain traps I could have escaped if they had talked to me….’ (male youth). P. 8-9. | Unequivocal |  |  |
| F70. Poor role models deterred risky sexual behavior reduction among female youth | *‘*When they [parents] fail to be good examples themselves, then what about children? They [parents] are marrying many wives…, seducing young people into sex… sugar daddies enticing young girls with gifts…’ (female youth). P. 9. | Unequivocal |  |  |
| F71. Restrictive parenting enabled risky sexual behavior reduction among female youth | ‘…for me, I never had a chance to get involved in sexual relationships because my father was very strict and could not allow me get out of the house or give me gap…’ (female youth). P. 10. | Unequivocal |  |  |
| 72. Lack of restrictive parenting impeded risky sexual behavior reduction among female youth | ‘……But when he died, I was like aha I am now free to get involved in sexual relationships.’ (female youth). P. 10. | Unequivocal |  |  |
| F73. Peer pressure hindered risky sexual behavior reduction among male youth | ‘…My friends tell me this, they tell me that, they discourage me from sex abstaining…..’ (male youth). P. 10. | Unequivocal |  |  |
| F74. Positive peer influence facilitated risky sexual behavior reduction among female youth | ‘…Her testimony encouraged me to be like her, so, for the boy friend I have now, we agreed to love each other without sex.’ (female youth). P. 10. | Unequivocal |  |  |
| F75. Gender-biased social norms hindered risky sexual behavior reduction among female youth | ‘You are trying to protect yourself but here people will call you a prostitute if they see you're a girl and you are buying or carrying condoms…...’ (female youth). P. 11. | Unequivocal |  |  |
| F76. Teacher advice promoted risky sexual behavior reduction among female youth | ‘…I decided to abstain because our teachers told us that a girl’s greatest gift she can give to her husband is virginity…..’ (female youth). P. 12. | Unequivocal |  |  |
| F77. Lack of financial support from family deterred risky sexual behavior reduction among female youth | ‘…but sometimes it [abstinence] becomes hard because I don’t have money and another thing is that I am an orphan; when my parents died it’s like I lost hope and I find myself into sex because of survival.’ (female participant). P. 12. | Unequivocal |  |  |
| F78. Religious beliefs discouraging risky sexual behavior promoted risky sexual behavior reduction among female youth | ‘…I can [abstain] because…it also goes against my Christian values to engage in sex before marriage….’ (female youth). P. 12. | Unequivocal |  |  |

**Additional file 2: Table S9: Data extraction from Ridgeway et al., 2020. ‘I married when I was 16… due to poverty, I had no other way’: multi-level factors influencing HIV-related sexual risk behaviours among adolescent girls in Zambézia, Mozambique**

| **Findings** | **Illustration** | **Evidence** | | |
| --- | --- | --- | --- | --- |
|  |  | **Unequivocal** | **Credible** | **Unsupported** |
| F79. Being self-reliant promoted risky sexual behavior reduction among youth | ‘….Men don’t fool around with me anymore because I have my own money….’ (Rabia). P. 6. | Unequivocal |  |  |
| F80. Parental advice facilitated risky sexual behavior reduction among youth. | ‘They say we don’t have to sleep with men for money: ‘You now have your own money. Make an effort to have much more to avoid pregnancy, HIV, and to continue to study.’ (Rabia). P. 7. | Unequivocal |  |  |
| F81. Having high self-motivation promoted risky sexual behavior reduction among youth | Five girls with ‘reduced risk’ were described as avoiding risk behaviours at Time 2 because they were particularly motivated to achieve their goals or determined to avoid HIV or pregnancy. P. 7. |  | Credible |  |
| F82. Negative experiences in a relationship enhanced risky sexual behavior reduction among youth | ‘Ma’am, I used to be with the father of my son all the time. [ … ] He used me, got me pregnant, and abandoned me with a child in my hands. I stopped going to school, and now I am here at home, not studying. Now I don’t date anybody.’ (Rabia, 18 years, Time 2). P. 8. | Unequivocal |  |  |
| F83. Partner’s refusal of condom use hindered risky sexual behavior reduction among youth | ‘My thoughts on condoms have not changed, I know that it’s for our protection against HIV/AIDS and other sexually transmitted diseases. Just my husband doesn’t accept it anymore, we used it 2 or 3 times and he doesn’t accept it anymore….’ (Yadira, 18 years, Time 2). P. 9. | Unequivocal |  |  |
| F84. Partner’s preferences and gender-biased norms hindered risky sexual behavior reduction among youth | Despite girls’ knowledge of and desire to use condoms, their use was limited by their husbands’ preferences and local norms against using condoms while married. P. 8-9. |  | Credible |  |
| F85. Gender-based violence hindered risky sexual behavior reduction among youth | Two girls experienced physical and emotional gender-based violence from their husbands; although both girls described these marriages as ones of love and mutual interest rather than financial necessity at Time 1, by Time 2 they and their salient references stated that the husbands had developed controlling behaviour and had ‘beaten’ and ‘insulted’ the girls. P. 9. |  | Credible |  |
| F86. Lack of financial support from family impeded risky sexual behavior reduction among female youth | ‘I got married before I was 18 years old because my father died … I’m very sad. My sister and I stopped studying because we don’t have anything, my mother is not able to buy clothes and notebooks because she also doesn’t have anything…..’ (Yadira, 18 years, Time 2). P. 10. | Unequivocal |  |  |
| F87. Having strong ambitions/being future-oriented promoted risky sexual behavior reduction among youth | ‘I don’t think about getting married early because I want to study first and only get married when I’m a grown up…’ (Abelina, 15 years, Time 1). P.11 | Unequivocal |  |  |
| F88. Financial support from family facilitated risky sexual behavior reduction among youth | ‘…..I still have my money to buy my school supplies, and even if I didn’t have money my parents would sell corn and beans to buy books for us.’ (Abelina, 15 years, Time 1). P.11. | Unequivocal |  |  |
| F89. Parental/family support promoted risky sexual behavior reduction among youth | Four girls with reduced risk described receiving significant support from their households specifically to continue with their education……... P. 11. |  | Credible |  |
| F90. Parental/family support promoted risky sexual behavior reduction among youth | …a cousin stated that both she and her father (the girl’s uncle) encouraged the girl to continue with school and stated that ‘Father tells her ‘You have to keep going to school, you can’t stop going to school’‘(Delma, Lidia’s cousin, head of household, adult, Time 2). P. 11. | Unequivocal |  |  |
| F91. Inaccessibility of services and cost of services hindered risky sexual behavior reduction among youth | …..distance to and cost of secondary education were often driving factors for engagement in sexual risk behaviour among girls with continued risk…. P.11. |  | Credible |  |

**Additional file 2: Table S10: Data extraction from Rohrbach et al., 2019. Effectiveness Evaluation of It's Your Game: Keep It Real, a Middle School HIV/Sexually Transmitted Infection/Pregnancy Prevention Program**

| **Findings** | **Illustration** |
| --- | --- |
| F92. Implementation of intervention with fidelity enhanced program success | Based on attendance data, we estimate a mean reach of 86.1% of the seventh- and eighth-grade students (n=50,766) enrolled in the participating schools across 3 years of program implementation. P. 385. |
| F93. Implementation of intervention with fidelity promoted program success | On overage, teachers reported completing delivery of 91% of the intended program lessons. P. 385. |
| F94. Implementation of intervention with fidelity promoted intervention success | On a scale ranging from 1=poor to 5=excellent, observers rated the quality of implementation as 4.25. P. 385. |

**Additional file 2: Table S11: Data extraction from Sales et al., 2012. Exploring factors associated with nonchange in condom use behavior following participation in an STI/HIV prevention intervention for African-American adolescent females**

| **Findings** | **Illustration** |
| --- | --- |
| F95. Having higher sensation seeking hindered risky sexual behavior reduction among youth | In a multivariate logistic regression model, the nonchange group was more likely to have (a) higher sensation seeking (AOR = .91, *P = .*023) |
| F96. Relationship issues (current boyfriend) impeded risky sexual behavior reduction among youth | In a multivariate logistic regression model, the nonchange group was more likely to have (b) a boyfriend (AOR = .32, *P = .*046) |
| F97. Violent partner hindered risky sexual behavior reduction among youth | In a multivariate logistic regression model, the nonchange group was more likely to have (c) a physical abuse history (AOR = .56, *P = .*057) |

**Additional file 2: Table S12: Data extraction from Sales et al., 2012a. Exploring why young African American women do not change condom-use behavior following participation in an STI/HIV prevention intervention**

| **Findings** | **Illustration** | **Evidence** | | |
| --- | --- | --- | --- | --- |
|  |  | **Unequivocal** | **Credible** | **Unsupported** |
| F98. Partner’s refusal of condom use hindered risky sexual behavior reduction among youth. | She indicates that her partner got upset when she mentioned wanting to use condoms for this reason. P. 1096. |  | Credible |  |
| F99. Relationships issues (unstable relationships) hindered risky sexual behavior reduction among youth | Having ‘on and off’ relationships, usually with the father of their child(ren) presents unique challenges to young women and may be a significant factor affecting their decision not to change condom use after AFIYA. P. 1096. |  | Credible |  |
| F100. Controlling partner deterred risky sexual behavior reduction among youth | ‘I was his girl, I was his property…. ‘[#103]). P. 1096. | Unequivocal |  |  |
| F101. Male partner suspects fidelity if a female partner request using a condom -impeded risky sexual behavior reduction among youth | ‘Oh man, you just with me. Why –what you got something to hide?’ (a young woman [#105]). P. 1097. | Unequivocal |  |  |
| F102. Partner’s refusal of condom use hindered risky sexual behavior reduction among youth | Additionally, they described their partners as not liking condoms, and having several complaints about their comfort. P. 1097. |  | Credible |  |
| F103. Desire for pregnancy and partner’s desire for pregnancy hindered risky sexual behavior reduction among youth | Several young women indicated either they or their male partner desired pregnancy, or they were not opposed to the woman becoming pregnant if it were to happen. P. 1097. |  | Credible |  |
| F104. Partner is/being under the influence of alcohol/drugs impeded risky sexual behavior reduction among youth | ‘But like one day we were like intoxicated, and we decided that we was gonna have a baby. And then after that, we just kept doing it [unprotected sex].’ (young woman [#106]). P. 1097. | Unequivocal |  |  |
| F105. Being stubborn/ hardheaded hindered risky sexual behavior reduction among youth | In addition to pregnancy, ‘non-changers’ frequently indicated they were incapable of change. Some referred to this as being stubborn or hardheaded. P. 1097. |  | Credible |  |
| F106. Lack of self-confidence impeded risky sexual behavior reduction among youth | ‘……..I would say that it’s just having that confidence to come to your partner and like – and just tell him everything because I’ve came a long way, but it’s like there’s still, you know, some things that I know or I notice [cheating] and I don’t say anything.’ (one participant [#106]). P. 1097. | Unequivocal |  |  |
| F107. Belief that one is incapable of change deterred risky sexual behavior reduction among youth | ‘I tried to change – I tried to be abstinent and I just kept getting pregnant. I just kept getting pregnant, I’m too fertile.’ (another young women [#107]). P. 1097. | Unequivocal |  |  |
| F108. Being reliant on avoidance strategies hindered risky sexual behavior reduction among youth | ‘And then I try to leave and he cry and do all that stuff. I don’t need no more stress until I deliver so . . .,’ (another young women [#107]). P. 1097. | Unequivocal |  |  |
| F109. Being unprepared deterred risky sexual behavior reduction among youth | ‘I wasn’t thinking about using nothing. So that – that’s a slipup and I kinda felt a little bad about that.’ (one young woman [#108]). P. 1098. | Unequivocal |  |  |
| F110. Fear of relationship breakdown impeded risky sexual behavior reduction among youth | ‘. . . I think I was scared to bring up that conversation to talk to him about it.’ (another young women [#109]). P. 1098. | Unequivocal |  |  |
| F111. Having self-confidence and having self-respect encouraged risky sexual behavior reduction among youth | She later describes how she eventually ‘gained the strength’ to talk to him about it, and how having respect for herself and self-confidence helped her. P. 1098. |  | Credible |  |
| F112. Being knowledgeable and having good problem-solving skills promoted risky sexual behavior reduction among youth | ‘Changers’ also appear to be more reflective, aware of, or more capable of recognizing negative partner characteristics and terminating unsatisfactory relationships. P.1098. |  | Credible |  |
| F113. Having high sense of responsibility facilitated risky sexual behavior reduction among youth | Additionally, when ‘slip ups’ occurred, they assumed responsibility for the situation.....P. 1098. |  | Credible |  |
| F114. Being under the influence of alcohol/drugs impeded risky sexual behavior reduction among youth | ‘…….So, I think that’s probably one of the main reasons why I got pregnant twice, from drinking…..’ (a young woman [#110]). P. 1098. | Unequivocal |  |  |
| F115. Low perceptions of risk of sexually transmitted infections including HIV deterred risky sexual behavior reduction among female youth | Another reason provided for non-condom use among the ‘changers’ was that they and their male partners (usually a long-term boyfriend) both were tested for STIs (usually together), so they felt it was ‘safe’ for them to have unprotected sex. P. 1098. |  | Credible |  |
| F116. Stable relationships promoted risky sexual behavior reduction among youth | Finally, overall, young women who were successful at changing their condom-use post-intervention rarely ever reported being in transitory or ‘on and off’ relationships. Instead, they typically described their on-going relationships as being ‘long-term’, trusting (on both sides), or ‘committed’. P. 1098. |  | Credible |  |
| F117. Partner’s consent of condom use and male partner does not suspect fidelity if a female partner request using a condom – promoted risky sexual behavior reduction among youth | They rarely indicated that their male partners complained about condom use or that their male partners would try to use the suggestion of condom use to question the young women’s fidelity. P. 1098 |  | Credible |  |

**Additional file 2: Table S13: Data extraction from Wamoyi et al., 2012. Recall, relevance and application of an in-school sexual and reproductive health intervention 7–9 years later: perspectives of rural Tanzanian young people**

| **Findings** | **Illustration** | **Evidence** | | |
| --- | --- | --- | --- | --- |
|  |  | **Unequivocal** | **Credible** | **Unsupported** |
| F118. Compatibility of intervention content with the needs of youth promoted intervention acceptability among youth | ‘ . . . all of them . . . . they were interesting to us in all aspects . . . you know, we’d be taught about how to protect oneself . . . so almost everything was advantageous.’ (a married male participant). P. 314. | Unequivocal |  |  |
| F119. Gender-biased norms, desire for pregnancy/children and economic constrains impeded risky sexual behavior reduction among female youth | Although they recalled some of the teachings such as those related to condom use and delaying sexual debut, they reported challenges to implementation such as cultural norms around masculinity and restrictions around female negotiation of sex, desire for children and economic constraints. P. 315. |  | Credible |  |
| F120. Lack of financial support from family hindered risky sexual behavior reduction among female youth | More than half of the young women (7/12) mentioned reasons for not abstaining before marriage such as lack of economic support from their families and hence reliance on sexual partners for their needs,…P. 315. |  | Credible |  |
| F121. Peer pressure hindered risky sexual behavior reduction among male youth | …men mentioned that they had been influenced by their peers to engage in sexual activity. P. 315. |  | Credible |  |
| F122. Being stubborn/ hardheaded impeded risky sexual behavior reduction among female youth | ‘. . . .Of course, you’d know indeed that there is a danger, but you’d just do it without a condom . . ..’ (a single young woman). P. 315. | Unequivocal |  |  |
| F123. Low perceptions of risk of sexually transmitted infections including HIV deterred risky sexual behavior among male youth | …..they said that they usually used condoms with women they did not trust and during a first sexual encounter with a new partner but not with women they trusted and/or planned to marry. P. 315. |  | Credible |  |
| F124. Fear of sexually transmitted infections including HIV promoted risky sexual behavior reduction among male youth | ‘With these ones I used [condoms] because I didn’t trust them a lot.’ (a married male participant). P. 315. | Unequivocal |  |  |
| F125. Fear of relationship breakdown hindered risky sexual behavior reduction among female youth | Many women reported finding it difficult to use condoms, citing barriers to use such as……fear about the consequences of requesting their partner to use a condom. P. 315. |  | Credible |  |
| F126. Partner’s consent of condom use promoted risky sexual behavior reduction among female youth | For the few women who reported ever having used a condom, they said that this was rare and only happened if their partners decided to use one. P. 315-6 |  | Credible |  |
| F127. Fear of relationship breakdown hindered risky sexual behavior reduction among female youth | Married females often felt unable to tell their spouse to use condoms, as this would be interpreted as their having been unfaithful. P. 316. |  | Credible |  |
| F128. Fear of getting sexually transmitted infections including HIV promoted risky sexual behavior reduction among female youth | ‘. . . He asked why I wanted him to use a condom and I pretended I was tired of giving birth but in reality, I feared AIDS as well as pregnancy.’ (a single woman). P. 316. | Unequivocal |  |  |
| F129. Preferring not to adopt an HIV prevention method (e.g., condom use) hindered risky sexual behavior reduction among female youth | ‘I just decided not to use them . . . I just preferred not use.’ (a married woman). P. 316. | Unequivocal |  |  |
| F130. Fear of pregnancy promoted risky sexual behavior reduction among female youth | The main motivations for pregnancy prevention were fear of consequences such as being sent away from school or their home. P. 316. |  | Credible |  |
| F131. Negative experiences associated with using contraceptives discouraged risky sexual behavior reduction among female youth | ‘……I tried using injectable contraception but stopped after only one round (3 months) because of bleeding continuously for one and a half months.’ (two single women). P. 316. | Unequivocal |  |  |
| F132. Desire for pregnancy/children impeded risky sexual behavior reduction among female youth | ‘. . . I said that, maybe let me try this one [having sex with a different man], maybe I will be lucky to get pregnant and get a baby . . .’ (one married woman). P. 317. | Unequivocal |  |  |
| F133. Controlling partner hindered risky sexual behavior reduction among female youth | When they started engaging in sex, their partners were very much in control of many decisions in their relationships, leaving those who wished to apply the intervention messages with limited opportunity to do so. P. 317. |  | Credible |  |
| F134. Gender-biased norms deterred risky sexual behavior reduction among female youth | Moreover, the women seemed to adhere more to the cultural expectations around gender power relations than to the intervention messages, some of which questioned those gender norms. P. 317. |  | Credible |  |
| F135. Incompatibility of intervention content with the needs of youth impeded intervention acceptability among male youth | One criticism of the MkV sessions from some male participants was that they were not linked closely enough to the long-term aspirations of the students. P. 317. |  | Credible |  |

**Additional file 2: Table S14: Data extraction from Wight et al., 2012. The need to promote behaviour change at the cultural level: one factor explaining the limited impact of the *MEMA kwa Vijana* adolescent sexual health intervention in rural Tanzania. A process evaluation**

| **Findings** | **Illustration** | **Evidence** | | |
| --- | --- | --- | --- | --- |
|  |  | **Unequivocal** | **Credible** | **Unsupported** |
| F136. Failure to implement the intervention with fidelity deterred program success | …participant observation suggested that many adults knew very little about the purpose and content of the intervention…P. 3. |  | Credible |  |
| F137. Incompatibility of intervention content with the needs of community members deterred intervention acceptability among community members. | …..and some themes, in particular condom use, were controversial. P. 3 |  | Credible |  |
| F138. Implementation of intervention with fidelity promoted program success | Process evaluation found that training courses for teachers, class peer educators, health workers and condom promoter-distributors were well implemented. Almost all teachers delivered most sessions…P. 3 |  | Credible |  |
| F139. Use of participatory facilitating methods, decreased corporal punishment promoted program success | …..some adopted interactive teaching styles and corporal punishment decreased. P. 3 |  | Credible |  |
| F140. Failure to implement the intervention with fidelity deterred program success | However, condoms could not be shown in primary school, leaving some pupils fundamentally confused…P. 3 |  | Credible |  |
| 141. Use of non-participatory facilitating methods, corporal punishment and sexual abuse impeded program success | ….and some teachers continued non-participatory teaching, corporal punishment, or, occasionally, sexual abuse. P. 3. |  | Credible |  |
| F142. Implementation of intervention with fidelity promoted program success | Class peer educators performed the drama serial well…. P.3. |  | Credible |  |
| F143. Implementers’ knowledge related to intervention content enhanced program success | Class peer educators… generally understood the intervention content much better than their classmates. P.3. |  | Credible |  |
| F144. Implementers’ lack of knowledge related to intervention content deterred program success | However, they could not answer complex questions…P.3. |  | Credible |  |
| F145. Being uncooperative/stubborn/hard headed deterred program success | …other pupils sometimes ignored or rejected their opinions. P.3. |  | Credible |  |
| F146. Implementation of intervention with fidelity facilitated program success | Most classes visited a health facility as recommended,…P. 3. |  | Credible |  |
| F147. Concern for privacy and fear of stigma hindered youth’s participation in the intervention | …participant observation suggested that adolescents remained very reluctant to use health services, fearing poor confidentiality and stigma. P. 3. |  | Credible |  |
| F148. Failure to implement the intervention with fidelity hindered program success and implementation of intervention with fidelity promoted program success | The stimulated patient visits showed that, for those who did, provision was variable and limited, but of higher quality than in control communities. P. 3. |  | Credible |  |
| F149. Failure to implement the intervention with fidelity deterred program success | Few pupils or other villagers had much awareness *of MEMA kwa Vijana* condom promoter-distributors…….. P. 3. |  | Credible |  |
| F150. Implementers’ lack of exemplary behavior hindered program success | Promoters reported difficulty selling condoms and were rumoured not to use condoms themselves, which was generally confirmed by their own accounts and those of their sexual partners. P. 3. |  | Credible |  |
| F151. Limited demand for services (e.g. condoms), cost of services (e.g. condoms) hindered program success | Due to the lack of demand for condoms, unsustainability, and cost, this intervention component was discontinued. P. 3 |  | Credible |  |
| F152. Women’s subordinate status and limited economic opportunities deterred risky sexual behavior reduction among youth | Women’s subordinate status and very limited economic opportunities were fundamental barriers to sexual behaviour change. P. 4. |  | Credible |  |
| F153. Desire to meet basic material needs impeded risky sexual behavior reduction among female youth | ‘What use is pleasure when there is no money?’ (18 year old woman). P. 4. | Unequivocal |  |  |
| F154. Peer pressure hindered risky sexual behavior reduction among female youth | A 14 year old school girl described how a girl would be treated who received nothing for sex: “They will shout at her. They will laugh at her. . . . those girls will sit in groups just gossiping about her, and she will have no comfort, she will stay lonely”. P. 4. | Unequivocal |  |  |
| F155. Partner’s refusal of condom use impeded risky sexual behavior reduction among female youth | Transactional sex…It also discouraged condom use because many men would refuse payment. P. 4. |  | Credible |  |
| F156. Limited intervention discouraged risky sexual behavior reduction among female youth | Although the MEMA kwa Vijana curriculum addressed transactional sex in several sessions, it did not recognise how fundamental the practice was both economically and culturally. P. 4. |  | Credible |  |
| F157. Poverty hindered program success | At a broader level, Tanzania’s poverty meant that the education and health services, particularly in remote rural regions, were of very poor quality. P.4 |  | Credible |  |
| F158. Poor education or training of implementers deterred program success | Teachers themselves were often poorly educated... P. 4 |  | Credible |  |
| F159. Having low literacy, having limited knowledge (e.g., sexual health and lack of support for critical thinking among youth hindered program success | Rural pupils had very low literacy, very limited knowledge of biology, and received little encouragement to think critically, hindering their engagement with *MEMA kwa Vijana*. P. 4. |  | Credible |  |
| F160. Limited resources (e.g. financial and human resources) deterred program success | Limited resources also meant health facilities often had inadequate staff training and chronic understaffing, with one-third of posts unfilled. P. 4. |  | Credible |  |
| F161. Inaccessibility of services (e.g. health centers), limited resources and poor quality of services (e.g., lack of confidentiality, inappropriate clinical advice) hindered program success | Health centers were typically 3-10 kilometres away from homes and reached by foot. They were frequently poorly stocked and clinical advice could be inappropriate and/or not confidential. P. 4. |  | Credible |  |
| F162. Having limited sexual health knowledge hindered risky sexual behavior reduction among female youth | Many young women were misinformed and/or appeared ambivalent about pregnancy. P. 4. |  | Credible |  |
| F163. Belief that one is incapable of change impeded risky sexual behavior reduction among female youth | Amongst those sexually active many believed conception was beyond their control, being determined by God or chance. P. 4. |  | Credible |  |
| F164. Fear of side effects of contraceptives deterred risky sexual behavior reduction among female youth | Amongst those who actively tried to prevent pregnancy, ineffective traditional methods were widespread, while hormonal contraception was feared to risk permanent infertility. P. 4. |  | Credible |  |
| F165. Myths about contraceptives hindered risky sexual behavior reduction among female youth | ‘……some are said to have a rotting uterus after using contraceptive pills or injections for a long time.’ (female informant). P.4. | Unequivocal |  |  |
| F166. Cultural beliefs hindered risky sexual behavior among youth | Their behaviour is unlikely to change if the beliefs of those around them not involved in the intervention, especially adults, remain unaffected. P. 5. |  | Credible |  |
| F167. Failure to implement the intervention with fidelity deterred program success | Those not at school had marginal exposure to *MEMA kwa Vijana*,..P. 5. |  | Credible |  |
| F168. Having limited knowledge (e.g., about the intervention) and being uncooperative deterred program success | …and many did not understand or support its goals. P. 5. |  | Credible |  |
| F169. Gender-biased norms deterred risky sexual behavior reduction among male youth | For all men, but particularly those not yet married or with children, sexual experience was a source of great pleasure, prestigious and central to their masculine identity. P. 5. |  | Credible |  |
| F170. Peer pressure impeded risky sexual behavior reduction among male youth | A 19 year old man explained he first had sex at 11: “due to the pressure he was getting from youth who already had begun having sex. . . ..” | Unequivocal |  |  |
| F171. Norms discouraging discussion of sexual issues between parents and children deterred risky sexual behavior among male youth | ‘Young people have to hide [relationships] … because after parents discover …, they’ll expect [the boy] to marry her or to give her a lot of money.’ (one field worker). P. 5. | Unequivocal |  |  |
| F172. Myths about contraceptives (condoms) hindered risky sexual behavior reduction among youth | While the most widely expressed belief was that condoms reduce men’s sexual pleasure, expressed in metaphors such as “eating a sweet in its wrapper”, they were also associated with infection and infidelity. P. 5. |  | Credible |  |
| F173. Norms discouraging discussion of sexual issues between parents and children impeded risky sexual behavior reduction among youth | Furthermore, adults opposed discussing condoms with young people in case of promoting sexual activity. P. 5. |  | Credible |  |
| F174. Inaccessibility of services (e.g., healthcare facilities), limited resources (e.g., condoms) and poor quality of services (e.g., lack of confidentiality) deterred risky sexual behavior reduction among female youth | For the rare young people who may have considered using condoms, access was limited, especially for girls, with intermittent supplies, distant health facilities, and limited confidentiality. P. 5. |  | Credible |  |
| F175. Restrictions on depicting of condoms impeded program success | However, educational restrictions on depicting condoms means many pupils and even peer educators only vaguely understood how they were used. P. 6. |  | Credible |  |
| F176. Low perceptions of risk of HIV infection discouraged risky sexual behavior reduction among youth | Villagers generally thought it affected those living in towns and cities, particularly those with “immoral” sexual practices, and adults rather than young people. P. 6. |  | Credible |  |
| F177. Poor decision-making skills hindered risky sexual behavior reduction among youth | In sexual encounters young people often focused on the immediate advantages of sex, rather than possible unwanted pregnancy or infection, even when these consequences were well known. P. 6 |  | Credible |  |
| F178. Norms encouraging healthy sexual practices promoted risky sexual behavior reduction among youth | The most important restrictive norms were that school pupils should be abstinent, young women should avoid overt premarital sex, married couples (particularly wives) should be faithful…….. P. 6. |  | Credible |  |
| F179. Restrictive parenting enabled risky sexual behavior reduction among female youth | Many parents were keen that pregnancy or early marriage should not waste the costs of their daughters’ schooling, and so they tried to prevent them until primary school was completed. P. 6. |  | Credible |  |
| F180. Parental/family support promoted risky sexual behavior reduction among female youth | The few young women at secondary school tended to have extraordinary parental support, creating a strong obligation to avoid pregnancy,… P. 6. |  | Credible |  |
| F181. Low socio-economic status promoted risky sexual behavior among male youth | …school boys generally had less money than their out-of-school peers to pay for sex. P. 6. |  | Credible |  |
| F182. Having strong ambitions/ being future-oriented promoted risky sexual behavior reduction among youth | More generally, the small minority of youth with strong educational ambitions were more averse to sexual risk, because they were more future-oriented. P. 6. |  | Credible |  |
| F183. Family/parental religious beliefs encouraged risky sexual behavior reduction among youth | Growing up in a devout Christian or Muslim family promoted abstinence and fidelity, especially if the young shared their parents’ strong religious beliefs. P. 6. |  | Credible |  |
| F184. Restrictive parenting promoted risky sexual behavior reduction among youth | In terms of sexual health, parental concern was primarily expressed through trying to prevent sexual activity. P. 6-7. |  | Credible |  |
