# Supplementary material for: Barriers and facilitators to HIV prevention interventions for reducing risky sexual behavior among youth worldwide: a systematic review
Source: BMC Infect Dis. 2022 Aug 8;22:679. doi: 10.1186/s12879-022-07649-z (PMC9361597; doi:10.1186/s12879-022-07649-z)
Supplement: Supplementary file 3 — Additional file 3. Table S1. Comparison of the identified barriers and facilitators between low/middle- and high-income countries. Table S2. Comparison of the identified barriers and facilitators between male and female youth [file 12879_2022_7649_MOESM3_ESM.docx]

**Additional file 3: Table S1: Comparison of the identified barriers and facilitators between low/middle- and high-income countries**

|  | |
| --- | --- |
| **Low/middle-income countries** | **High-income countries** |
| **Barriers** | |
| **1. Characteristics of the implementation object**  1.1 Barriers to intervention acceptability among youth   - Long duration of the intervention [33] - Incompatibility of intervention content with the needs of youth [33, 43] - Not reported   1.2 Barriers to intervention acceptability among community members   - Incompatibility of intervention content with the needs of community members [44]   1.3 Barriers to youth’s participation in the intervention   - Age requirements that excluded other youth [33] - Not reported   1.4 Barriers to risky sexual behavior reduction among youth   - Limited intervention content (e.g., intervention content addresses individual factors such as knowledge without addressing structural factors such as poverty and unemployment) [32, 44] | **1. Characteristics of the implementation object**  1.1 Barriers to intervention acceptability among youth   - Long duration of the intervention [35] - Not reported - Complexity of the intervention [35]   1.2 Barriers to intervention acceptability among community members   - Not reported   Barriers to youth’s participation in the intervention   - Not reported - Restricted days and times of the intervention [34]   1.4 Barriers to risky sexual behavior reduction among youth   - Not reported |
| **2. Characteristics of the users/adopters**  2.1 Barriers to intervention acceptability among youth   - Not reported   2.2 Other barriers   - Implementers’ lack of knowledge related to intervention content [44] - Poor education or training of implementers [44] - Implementers’ lack of exemplary or positive behavior [44] | **2. Characteristics of the users/adopters**  2.1 Barriers to intervention acceptability among youth   - Adult/old implementers [37]   2.2 Other barriers   - Not reported - Not reported - Not reported |
| **3. Characteristics of the end users**  3.1 Barriers to risky sexual behavior reduction among youth   - Low perceptions of risk of sexually transmitted infections including HIV [38, 43, 44] - Fear of relationship breakdown [33, 38, 43] - Desire for pregnancy/children [43] - Being stubborn/hard hardheaded [43] - Belief that one is incapable of change [44] - Negative attitudes towards condom use [32, 44] - Poor decision-making skills [44] - Concern for privacy [33] - Fear of side effects of contraceptives [44] - Preferring not to adopt an HIV prevention method (e.g., condom use [43] - Limited sexual health knowledge [44] - Negative experiences associated with using contraceptives [43] - Desire to meet basic material needs [44] - Not reported - Not reported - Not reported - Not reported - Not reported   3.2 Barriers to youth’s participation in the intervention   - Concern for privacy [32, 44] - Fear of stigma [44]   3.3 Other barriers to intervention success   - Being stubborn/hardheaded/uncooperative [44] - Having limited knowledge (e.g., about the intervention [44] - Low literacy [44] | **3. Characteristics of the end users**  3.1 Barriers to risky sexual behavior reduction among youth   - Low perceptions of risk of sexually transmitted infections including HIV [42] - Fear of relationship breakdown [42] - Desire for pregnancy/children [42] - Being stubborn/hard hardheaded [42] - Belief that one is incapable of change [42] - Not reported - Not reported - Not reported - Not reported - Not reported - Not reported - Not reported - Not reported - Lack of self-confidence [42] - Having high sensation seeking [41] - Being under the influence of alcohol/drugs [42] - Being reliant on avoidance strategies [42] - Being unprepared [42]   3.2 Barriers to youth’s participation in the intervention   - Not reported - Not reported   3.3 Other barriers to intervention success   - Not reported - Not reported - Not reported |
| **4. Characteristics of the context**  4.1 Interpersonal  4.1.1 Barriers to risky sexual behavior reduction among youth   - Partner’s refusal to use an HIV prevention method (e.g., condom use, HIV testing [32, 33, 38, 39] - Controlling partner [43] - Violent partner [36] - Negative peer influence [38, 43, 44] - Lack of financial support from the family [38, 39, 43] - Lack of child-parent communication on sexual issues [36, 38] - Parent’s refusal of an HIV prevention method (e.g., HIV testing [32] - Partner’s negative attitudes towards condom use [38] - Poor role models [38] - Lack of restrictive parenting [38] - Partner’s preferences not to adopt an HIV prevention method (e.g., condom use [39] - Not reported - Peer pressure [44] - Not reported - Not reported - Not reported   4.1.2 Other barriers to intervention success   - Lack of support for critical thinking among youth [44]   4.2 Community  4.2.1 Barriers to risky sexual behavior reduction among youth   - Gender-biased norms [36, 38, 39, 43, 44] - Myths about contraceptives [32, 44] - Norms discouraging discussion of sexual issues between parents and children [32, 44] - Limited resources/services in the community (e.g. programs for youth, condoms) [32, 33] - Cultural beliefs [44]   4.2.2 Barriers to youth’s participation in the intervention   - Not reported - Not reported   4.3 Organizational or institutional  4.3.1 Barriers to risky sexual behavior reduction among youth   - Limited resources (e.g., condoms, human resources) [44] - Inaccessibility of services (e.g., condoms, healthcare facilities) [44] - Poor quality of services (e.g., lack of confidentiality) [44]   4.3.2 Barriers to youth’s participation in the intervention   - Not reported   4.3.3 Other barriers to intervention success   - Limited resources (e.g., financial and human resources) [44] - Restrictions on depicting of condoms in schools [44] - Poor quality of services (e.g. lack of confidentiality, inappropriate clinical advice) [44] - Inaccessibility of services (e.g., healthcare facilities [44]   4.4 Structural  4.4.1 Barriers to risky sexual behavior reduction among youth   - Economic constrains [32, 43] - Poverty [32] - Unemployment [32] - Limited economic opportunities [44] - Women’s subordinate status [44] - Cost of services (e.g. secondary education) [39] - Inaccessibility of services (e.g. schools) [39] - Gender-based violence [39]   4.4.2 Other barriers to intervention success   - Poverty [44] - Limited demand for services (e.g., condoms) [44] - Cost of services (e.g., condoms) [44] | **4. Characteristics of the context**  4.1 Interpersonal  4.1.1 Barriers to risky sexual behavior reduction among youth   - Partner’s refusal to use an HIV prevention method (e.g., condom use, HIV testing [42] - Controlling partner [42] - Violent partner [41] - Not reported - Not reported - Not reported - Not reported - Not reported - Not reported - Not reported - Not reported - Relationship issues (e.g., current boyfriend and unstable relationships) [41, 42] - Not reported - Male partner suspect fidelity if a female partner request protected sex [42) - Partner’s desire for pregnancy [42] - Partner is under the influence of drugs [42]   4.1.2 Other barriers to intervention success   - Not reported   4.2 Community  4.2.1 Barriers to risky sexual behaviour reduction among youth   - Not reported - Not reported - Not reported - Not reported - Not reported   4.2.2 Barriers to youth’s participation in the intervention   - Violence in the community/neighborhood [34] - Incarceration [34]   4.3 Organizational or institutional  4.3.1 Barriers to risky sexual behavior reduction among youth   - Not reported - Not reported - Not reported   4.3.2 Barriers to youth’s participation in the intervention   - Inaccessibility of intervention venue [34, 37]   4.3.3 Other barriers to intervention success   - Not reported - Not reported - Not reported - Not reported   4.4 Structural  4.4.1 Barriers to risky sexual behaviour among youth   - Not reported - Not reported - Not reported - Not reported - Not reported - Not reported - Not reported - Not reported   4.4.2 Other barriers to intervention success   - Not reported - Not reported - Not reported |
| **5. Characteristics of the strategy of facilitating implementation**  5.1 Barriers to intervention acceptability among youth   - Use of non-participatory facilitating methods) [32]   5.2 Other barriers to intervention success   - Failure to implement the intervention with fidelity [44] - Use of non-participatory teaching/facilitating methods [44] - Use of corporal punishment [44] - Sexual abuse [44] | **5. Characteristics of the strategy of facilitating implementation**  5.1 Barriers to intervention acceptability among youth   - Not reported   5.2 Other barriers to intervention success   - Not reported - Not reported - Not reported - Not reported |
| **Facilitators** | |
| **1. Characteristics of the implementation object**  1.1 Facilitators to intervention acceptability among youth   - Compatibility of intervention content with the needs of youth [36]   1.2 Facilitators to intervention acceptability among implementers   - Relative advantage of the intervention [32] | **1. Characteristics of the implementation object**  1.1 Facilitators to intervention acceptability among youth   - Compatibility of intervention content with the needs of youth [35, 37]   1.2 Facilitators to intervention acceptability among implementers   - Not reported |
| **2. Characteristics of the users/adopters**  2.1 Facilitators to intervention acceptability among youth   - Approachability/friendliness of implementers [33] - Not reported   2.2 Other facilitators to intervention success   - Training of implementers [32] - Implementers’ knowledge related to intervention content [44] | **2. Characteristics of the users/adopters**  2.1 Facilitators to intervention acceptability among youth   - Approachability/friendliness of implementers [37] - Experience of implementers [37]   2.2 Other facilitators to intervention success   - Not reported - Not reported |
| **3. Characteristics of the end users**  3.1 Facilitators to risky sexual behavior reduction among youth   - Fear of pregnancy/sexually transmitted infections including HIV [32, 43] - Having strong ambitions/being future oriented [39, 44] - Intentions/readiness to change [36] - Negative experiences in a relationship [39] - Being self-reliant [39] - Having high self-motivation [39 - Low socio-economic status (e.g., lack of money to pay for sex) [44] - Not reported - Not reported - Not reported - Not reported - Not reported   3.2 Facilitators to youth’s participation in the intervention   - Not reported | **3. Characteristics of the end users**  3.1 Facilitators to risky sexual behavior reduction among youth   - Fear of pregnancy/sexually transmitted infections including HIV [37] - Not reported - Not reported - Not reported - Not reported - Not reported - Not reported - Being knowledgeable [42] - Having good problem-solving skills [42] - Having high self-confidence [42] - Having high self-respect [42] - Having high sense of responsibility [42]   3.2 Facilitators to youth’s participation in the intervention   - Perceived benefits of the intervention [37] |
| **4. Characteristics of the context**  4.1 Interpersonal  4.1.1 Facilitators to risky sexual behavior reduction among youth   - Partner’s consent to adopt an HIV prevention method (e.g., condom use [43] - Family support [39, 44] - Restrictive parenting [38, 44] - Positive peer influence [38] - Teacher advice [38] - Parental advice [39] - Family/parental religious beliefs (e.g., raised in a family with religious beliefs against risky sexual behavior [44] - Not reported - Not reported   4.2 Community  4.2.1 Facilitators to risky sexual behavior reduction among youth   - Norms encouraging healthy sexual behavior (e.g., abstinence and delaying of sexual debut) [44] - Religious beliefs discouraging risky sexual behavior [38]   4.3 Organizational or institutional  4.3.1 Facilitators to intervention acceptability among youth   - Accessibility and friendliness of the intervention venue [33] | **4. Characteristics of the context**  4.1 Interpersonal  4.1.1 Facilitators to risky sexual behavior reduction among youth   - Partner’s consent to adopt an HIV prevention method (e.g., condom use [42] - Not reported - Not reported - Not reported - Not reported - Not reported - Not reported - Stable relationships - Partner does not suspect fidelity if the other partner request protected sex [42]   4.2 Community  4.2.1 Facilitators to risky sexual behaviour among youth   - Not reported - Not reported   4.3 Organizational or institutional  4.3.1 Facilitators to intervention acceptability among youth   - Not reported |
| **5. Characteristics of the strategy of facilitating implementation**  5.1 Facilitators to intervention acceptability among youth   - Use of same sex youth group [36] - Not reported - Not reported   5.2 Facilitators to youth’s participation in interventions   - Mobilization of community members to influence youth to attend the intervention [32] - Integration of intervention with other services [32] - Provision of detailed intervention information to parents [32] - Using outreach activities [32] - Building of a trusting relationship with young people [32] - Use of same age or peer implementers [32] - Provision of incentives [32]   5.3 Other facilitators to intervention success   - Dissemination of intervention information to community members [32, 36] - Implementation of intervention with fidelity [44] - Use of participatory facilitating methods [44] - Decreased corporal punishment [44] - Collaboration among different stakeholders in delivering the intervention [32] | **5. Characteristics of the strategy of facilitating implementation**  5.1 Facilitators to intervention acceptability among youth   - Use of same sex youth group [37] - Use of different or mixed facilitating methods [35] - Implementation of intervention with fidelity [37]   5.2 Facilitators to youth’s participation in interventions   - Not reported - Not reported - Not reported - Not reported - Not reported - Not reported - Not reported   5.3 Other facilitators to intervention success   - Dissemination of intervention information to community members [37] - Implementation of intervention with fidelity [40] - Not reported - Not reported - Not reported |

**Additional file 3: Table S2: Comparison of the identified barriers and facilitators between male and female youth**

| **Males** | **Females** |
| --- | --- |
| **Barriers** | |
| **1. Characteristics of the implementation object**  Barriers to intervention acceptability among youth   - Incompatibility of intervention content with the needs of youth [33, 43] - Long duration of the intervention [33, 35] - Complexity of the intervention [35]   Barriers to youth’s participation in the intervention   - Age requirements that excluded other youth [33] - Not reported   Barriers to risky sexual behavior reduction among youth   - Not reported | **1. Characteristics of the implementation object**  Barriers to intervention acceptability among youth   - Not reported - Not reported - Not reported   Barriers to youth’s participation in the intervention   - Not reported - Restricted days and times of the intervention [34]   Barriers to risky sexual behavior behavior among youth   - Limited intervention content (e.g., intervention content addresses individual factors such as knowledge without addressing structural factors such as poverty and unemployment) [32, 44] |
| **2. Characteristics of the users/adopters**  Barriers to intervention acceptability among youth   - Not reported | **2. Characteristics of the users/adopters**  Barriers to intervention acceptability among youth   - Adult/old implementers [37] |
| **3. Characteristics of the end users**  Barriers to risky sexual behavior reduction among youth   - Low perceptions of risk of sexually transmitted infections including HIV [33, 38, 43, 44] - Fear of relationship breakdown [33] - Poor decision-making skills [44] - Not reported - Not reported - Not reported - Not reported - Not reported - Not reported - Not reported - Not reported - Not reported - Not reported - Not reported - Not reported - Not reported - Not reported - Concern for privacy [33]   Barriers to youth’s participation in the intervention   - Concern for privacy [44] - Fear of stigma [44] | **3. Characteristics of the end users**  Barriers to risky sexual behavior reduction among youth   - Low perceptions of risk of sexually transmitted infections including HIV [42, 44] - Fear of relationship breakdown [38, 42, 43] - Poor decision-making skills [44] - Negative attitudes towards condom use [32] - Desire for pregnancy/children [42, 43] - Being stubborn/hard hardheaded [42, 43] - Belief that one is incapable of change [42, 44] - Lack of self-confidence [42] - Having high sensation seeking [41] - Being under the influence of alcohol/drugs [42] - Being reliant on avoidance strategies [42] - Being unprepared [42] - Fear of side effects of contraceptives [44] - Preferring not to adopt an HIV prevention method (e.g., condoms use) [43] - Limited sexual health knowledge [44] - Negative experiences associated with using contraceptives [43] - Desire to meet basic material needs [44] - Not reported   Barriers to youth’s participation in the intervention   - Concern for privacy [32, 44] - Fear of stigma [44] |
| **4. Characteristics of the context**  4.1 Interpersonal  Barriers to risky sexual behavior reduction among youth   - Partner’s refusal to use an HIV prevention method (e.g., condom use, HIV testing [33] - Lack of child-parent communication on sexual issues [38] - Peer pressure [38, 43, 44] - Not reported - Not reported - Not reported - Not reported - Not reported - Not reported - Not reported - Not reported - Not reported - Not reported - Not reported - Not reported   4.2 Community  Barriers to risky sexual behavior reduction among youth   - Gender-biased norms [44] - Myths about contraceptives [44] - Norms discouraging discussion of sexual issues between parents and children [44] - Limited resources/services in the community (e.g. programs for youth, condoms) [33] - Cultural beliefs [44]   Barriers to youth’s participation in the intervention   - Not reported - Not reported   4.3 Organizational or institutional  Barriers to risky sexual behavior reduction among youth   - Not reported - Not reported - Not reported   Barriers to youth’s participation in the intervention   - Not reported   4.4 Structural  Barriers to risky sexual behavior reduction among youth   - Not reported - Not reported - Not reported - Not reported - Not reported - Not reported - Not reported - Not reported | **4. Characteristics of the context**  4.1 Interpersonal  Barriers to risky sexual behavior reduction among youth   - Partner’s refusal to use an HIV prevention method (e.g., condom use, HIV testing [32, 38, 39, 42] - Lack of child-parent communication on sexual issues [36] - Peer pressure [44] - Lack of financial support from the family [38, 39, 43] - Controlling partner [42, 43] - Relationship issues (e.g., current boyfriend and unstable relationships) [41, 42] - Parent’s refusal of an HIV prevention method (e.g., HIV testing [32] - Violent partner [36, 41] - Partner’s negative attitudes towards condom use [38] - Poor role models [38] - Lack of restrictive parenting [38] - Partner suspect fidelity if the other partner request protected sex [42) - Partner’s desire for pregnancy [42] - Partner is under the influence of drugs [42] - Partner’s preferences not to adopt an HIV prevention method (e.g., condom use) [39]   4.2 Community  Barriers to risky sexual behavior reduction among youth   - Gender-biased norms [36, 38, 39, 43] - Myths about contraceptives [32, 44] - Norms discouraging discussion of sexual issues between parents and children [32, 44] - Limited resources/services in the community (e.g. programs for youth, condoms) [32] - Cultural chiefs [44]   Barriers to youth’s participation in the intervention   - Violence in the community/neighborhood [34] - Incarceration [34]   4.3 Organizational or institutional  Barriers to risky sexual behavior reduction among youth   - Limited resources (e.g., condoms, human resources) [44] - Inaccessibility of services (e.g., condoms, healthcare facilities) [44] - Poor quality of services (e.g., lack of confidentiality) [44]   Barriers to youth’s participation in the intervention   - Inaccessibility of intervention venue [34, 37]   4.4 Structural  Barriers to risky sexual behavior reduction among youth   - Economic constrains [32, 43] - Poverty [32] - Unemployment [32] - Limited economic opportunities [44] - Women’s subordinate status [44] - Cost of services (e.g. secondary education) [39] - Inaccessibility of services (e.g. schools) [39] - Gender-based violence [39] |
| **5. Characteristics of the strategy of facilitating implementation**  Barriers to intervention acceptability among youth   - Not reported | **5. Characteristics of the strategy of facilitating implementation**  Barriers to intervention acceptability among youth   - Use of non-participatory facilitating methods) [32] |
| **Facilitators** | |
| **1. Characteristics of the implementation object**  Facilitators to intervention acceptability among youth   - Compatibility of intervention content with the needs of youth [35, 36] | **1. Characteristics of the implementation object**  Facilitators to intervention acceptability among youth   - Compatibility of intervention content with the needs of youth [36, 37] |
| **2. Characteristics of the users/adopters**  Facilitators to intervention acceptability among youth   - Approachability/friendliness of implementers [33] - Not reported | **2. Characteristics of the users/adopters**  Facilitators to intervention acceptability among youth   - Approachability/friendliness of implementers [37] - Experience of implementers [37] |
| **3. Characteristics of the end users**  Facilitators to risky sexual behavior reduction among youth   - Fear of pregnancy/sexually transmitted infections including HIV [43] - Having strong ambitions/being future oriented [44] - Not reported - Not reported - Not reported - Not reported - Not reported - Not reported - Not reported - Not reported - Not reported - Low socio-economic status (e.g., lack of money to pay for sex) [44]   Facilitators to youth’s participation in the intervention   - Not reported | **3. Characteristics of the end users**  Facilitators to risky sexual behavior reduction among youth   - Fear of pregnancy/sexually transmitted infections including HIV [32, 37, 43] - Having strong ambitions/being future oriented [39, 44] - Being knowledgeable [42] - Having good problem-solving skills [42] - Having high self-confidence [42] - Negative experiences in a relationship [39] - Being self-reliant [39] - Having high self-motivation [39] - Having high self-respect [42] - Having high sense of responsibility [42] - Intentions/readiness to change [36] - Not reported   Facilitators to youth’s participation in the intervention   - Perceived benefits of the intervention [37] |
| **4. Characteristics of the context**  4.1 Interpersonal  Facilitators to risky sexual behavior reduction among youth   - Not reported - Not reported - Not reported - Not reported - Not reported - Not reported - Not reported - Not reported - Family/parental religious beliefs (e.g., raised in a family with religious beliefs against engaging in risky sexual behavior) [44]   4.2 Community  Facilitators to risky sexual behavior reduction among youth   - Norms encouraging healthy sexual behavior (e.g., abstinence and delaying of sexual debut) [44] - Not reported   4.3 Organizational or institutional  Facilitators to intervention acceptability among youth   - Accessibility and friendliness of the intervention venue [33] | **4. Characteristics of the context**  4.1 Interpersonal  Facilitators to risky sexual behavior reduction among youth   - Partner’s consent to use an HIV prevention method (e.g., condom use [42, 43] - Family support [39, 44] - Restrictive parenting [38, 44] - Positive peer influence [38] - Teacher advice [38] - Parental advice [39] - Stable relationships [42] - Partner does not suspect fidelity if the other partner request protected sex [42] - Family/parental religious beliefs (e.g., raised in a family with religious beliefs against engaging in risky sexual behavior) [44]   4.2 Community  Facilitators to risky sexual behavior reduction among youth   - Norms encouraging healthy sexual behavior (e.g., abstinence and delaying of sexual debut) [44] - Religious beliefs discouraging risky sexual behavior [38]   4.3 Organizational or institutional  Facilitators to intervention acceptability among youth   - Not reported |
| **5. Characteristics of the strategy of facilitating implementation**  Facilitators to intervention acceptability among youth   - Use of same sex youth group [36] - Use of different or mixed facilitating methods [35] - Not reported   Facilitators to youth’s participation in the intervention   - Mobilization of community members to influence youth to attend the intervention [32] - Integration of intervention with other services [32] - Provision of detailed intervention information to parents [32] - Using outreach activities [32] - Not reported - Not reported - Not reported | **5. Characteristics of the strategy of facilitating implementation**  Facilitators to intervention acceptability among youth   - Use of same sex youth group [36, 37] - Not reported - Implementation of intervention with fidelity [37]   Facilitators to youth’s participation in the intervention   - Mobilization of community members to influence youth to attend the intervention [32] - Integration of intervention with other services [32] - Provision of detailed intervention information to parents [32] - Using outreach activities [32] - Building of a trusting relationship with young people [32] - Use of same age or peer implementers [32] - Provision of incentives [32] |
